# Oral human papillomavirus infection aligns with a coordinated bacterial microbiome inferred virulence ecology

**DOI:** 10.3389/fcimb.2026.1821266

**Published:** 2026-06-05

**Authors:** Ramadhani Chambuso, Sara Alajrami, Nazia Wali Jan, Mushal Allam, Vijay Desai, Yehia S. Mohamed, Farah Al-Marzooq

**Affiliations:** 1 College of Medicine, Ajman University, Ajman, United Arab Emirates; 2 Department of Global Health and Population, Harvard T.H. Chan School of Public Health, Boston, MA, United States; 3 Ajman University, Ajman, United Arab Emirates; 4 Department of Genetics and Genomics, College of Medicine and Health Sciences, United Arab Emirates University, Al Ain, United Arab Emirates; 5 Department of Pathological Sciences, College of Medicine, Ajman University, Ajman, United Arab Emirates; 6 Department of Microbiology and Immunology, Faculty of Pharmacy (Boys), Al-Azhar University, Cairo, Egypt; 7 Department of Medical Microbiology and Immunology, College of Medicine and Health Sciences, United Arab Emirates University, Al Ain, United Arab Emirates

**Keywords:** bacterial genomics, microbiome virulence ecology, oral bacterial microbiome, oral ecosystem, oral HPV infection

## Abstract

**Background:**

The biological relationship between oral human papillomavirus (HPV) infection and the community-level virulence ecology of the oral bacterial microbiome remains unresolved due to taxon-centric analyses. We profiled non-cancer oral HPV infection status and the ecological virulence architecture of the oral bacterial microbiome by integrating with bacterial genomics.

**Methods:**

We used publicly available 16S rRNA gene sequencing data of the oral bacterial microbiome from 127 participants. Raw sequencing reads were quality filtered, denoised and taxonomically assigned at the genus level using standard amplicon-processing pipelines. Oral HPV status was derived from the original study metadata. Microbiome structure was characterised using diversity metrics, unsupervised ecotype and multivariate analyses. To interrogate functional organisation, bacterial genera were mapped to curated virulence-associated domains from published bacterial genomics databases. Variance partitioning was performed using PERMANOVA analysis to assess the independent contributions of HPV status and virulence ecology.

**Results:**

We observed a gross overlap across oral HPV groups (64 HPV-negative and 63 HPV-positive) with no dominant bacterial microbiome taxa after multiple-testing correction (all FDR > 0.10). Alpha diversity was modestly higher in HPV-positive samples, but differences were not statistically significant (Median difference = 0.18; 95% CI 0.05-0.41, p = 0.12). Unsupervised ecotype analysis identified three independent bacterial microbiome states, none defined by HPV status (All p > 0.20). In the multivariate PERMANOVA analysis, microbial diversity (R² = 8.9%, p < 0.001) and virulence ecology (R² = 3.9%, p < 0.001) explained significantly more community bacterial microbiome variance than HPV status (R² = 0.9%, p = 0.24). The structured network-level reprogramming of taxa and genera virulence ecology by HPV status showed almost similar genus-virulence coordination, particularly within adhesion, invasion and immune-interface modules and no significant differences in the mean virulence module abundance (all Cliff’s δ < 0.15). HPV-positivity aligned more with high virulence-pressure, low terrain of the oral bacterial microbiome ecological landscapes. Relative to the protected terrain (Q1), HPV positivity was more frequent in the higher-pressure ecological states, with odds ratios of 3.62 for Q2 (95% CI 1.19-11.06; p=0.024), 3.13 for Q3 (95% CI 1.02-9.58; p=0.045), and 3.68 for Q4 (95% CI 1.06-12.77; p=0.040). The strongest point estimate was observed in the low-diversity, high-pressure danger zone (Q4).

**Conclusions:**

In this hypothesis generation study, oral HPV infection aligns with coordinated inferred virulence ecology of the oral bacterial microbiome rather than discrete taxonomic or abundance-based changes.

## Graphical Abstract

**Figure:**
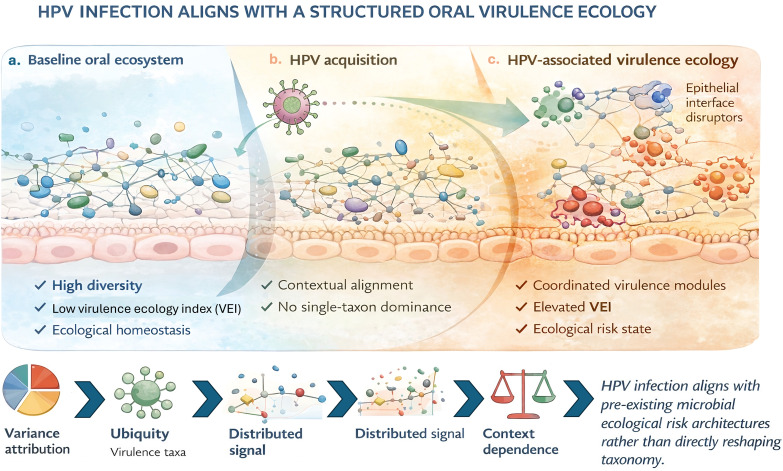

## Highlights

- Oral HPV infection reorganizes within the oral bacterial microbiome-inferred virulence ecology network.
- The inferred Virulence ecology organisation rewired across taxa without increasing relative abundancy.
- Targeting virulence ecology patterns may be more impactful than trying to eliminate specific taxa.

## Introduction

Despite extensive investigations, the biological relationship between oral human papillomavirus (HPV) infection and the virulence ecology of oral bacterial microbiome remains poorly resolved (Shigeishi et al., 2021; Dahlstrom et al., 2023; Zhang et al., 2024). Existing studies report inconsistent and often irreproducible taxonomic associations. Largely because previous analyses have focused on individual bacterial taxa or diversity metrics rather than community-level ecological organisation in the oropharynx (Yost et al., 2018; Zhang et al., 2022; Escobar Marcillo et al., 2025). This has obscured where the true microbiological signal of oral HPV infection resides and limited mechanistic interpretations exist. There is a critical gap in understanding whether oral HPV infection aligns with specific bacterial microbiome virulence ecology (Maidment et al., 2025; Renu, 2025; Bulfoni et al., 2026). Also, the functional architectures that govern epithelial colonisation, inflammation and host-microbe interactions at the microbial genomics level (Luca et al., 2018; Ma et al., 2024; Lawing and Bleich, 2025). Addressing this gap is essential to reconcile conflicting basic findings in the literature and establish a strong biologically coherent framework linking oral HPV infection with microbiome genomics (Zhang et al., 2022; Constantin et al., 2023).

We previously shown the molecular genetics effect of persistence high-risk HPV infection on carcinogenesis (Chambuso et al., 2018; Chambuso et al., 2019; Chambuso et al., 2020). However, oral HPV infection occurs within a complex oral microbial ecosystem. This directly interfere with stratified epithelium in the oropharynx mucosa, mucosal immunity and mucosal barrier biology (Radaic and Kapila, 2021; Salem, 2025). In persistent infection, high-risk HPV get access to the basal epithelial layer of the oropharynx, typically following micro abrasions. The viral oncoproteins E6 and E7 disrupt p53 and Rb tumour-suppressor gene pathways, enabling uncontrolled epithelial proliferation for carcinogenesis (Lechner et al., 2022; Zhang et al., 2025). HPV infection clearance depends on intact mucosal immunity type I/III interferons, epithelial barrier integrity, antimicrobial peptides and balanced microbiomes (Britto et al., 2020; Zhuoyao et al., 2025). Microbiome genomics has shown that stable colonisation and disease risk are rarely dictated by single organisms. Rather it can be detected by community organisation, functional redundancy and ecological virulence (Lamont et al., 2018; Boyraz et al., 2022). Yet most oral HPV studies have interrogated taxonomic level differences. Ecological virulence can restructure bacterial communities directly and permit epithelial access, immune evasion and favour long-term coexistence (Sulieman and Abdallah, 2024). Periodontal and anaerobic bacteria encode conserved virulence programs that modulate adhesion, invasion and host-microbe signalling. These functions are ecologically coordinated across taxa rather than confined to specific species. By integrating bacterial genomics with ecological virulence, we can test whether oral HPV infection maps onto higher-order microbiome virulence architectures (Hajishengallis and Lamont, 2021; Ren et al., 2024).

HPV infection persistence relies on subtle modulation of the epithelial-immune interface rather than tissue destruction (Lo Cigno et al., 2024). Such persistence is unlikely to depend on single bacterial species. Instead, it is more plausibly shaped by microbial communities that organise functions enabling adhesion, invasion and immune signalling at the mucosal surface (Li et al., 2022; Hajishengallis et al., 2023). Oral bacterial microbiomes share virulence programs that are distributed across taxa and expressed through coordinated ecological interactions. These programs can alter mucosal immunity and immune tone without changing overall bacterial abundance (Hajishengallis et al., 2023; Zhou et al., 2025). We hypothesised that oral HPV infection aligns with ecological virulence microbial communities that are coordinated, rather than driving direct compositional restructuring at the relative abundance level (Torralba et al., 2021; Adekoya et al., 2025; Jiang et al., 2025; Lin et al., 2025).

The aim of this study was to test whether oral HPV positivity in a non-cancer cohort is associated with community-level virulence ecology of the oral bacterial microbiome, rather than with discrete abundance shifts of individual taxa by integrating with bacterial genomics.

## Methods

### Ethics approval

This study used publicly available secondary metagenomic data obtained from the NCBI Sequence Read Archive (https://www.ncbi.nlm.nih.gov/bioproject/?term=PRJNA1178730, BioProject ID: PRJNA1178730) (Maidment et al., 2025). As all data are fully de-identified and accessible in the public domain, no direct interaction with human participants occurred and no identifiable personal information was used. In accordance with the principles of the Declaration of Helsinki, the study ensures respect for participant autonomy, privacy, and data protection by relying solely on anonymised datasets generated under prior ethical approval by the original investigators. Because this project involves secondary analysis of existing, non-identifiable data, additional institutional ethics approval was not required (Parums, 2024).

### Data source and study design

We used PRISMA workflow (shared in **Supplementary Material**) for screening and eligibility assessment to identify publicly available oral microbiome sequencing datasets suitable for secondary genomic analysis of HPV-microbiome virulence ecology. Although multiple studies reported oral microbiome data and HPV infection status, only one dataset met all predefined inclusion criteria, including adult participants, availability of sample-level oral HPV status, access to raw sequencing data and sufficient metadata for ecological and virulence-based analyses. This systematic approach ensured transparent dataset selection and minimised selection bias.

A cross-sectional study design was conducted by re-analysis of publicly available data. We used raw 16S rRNA full length sequencing from V1-V9 amplicon sequencing reads by downloading FASTQ files and accompanying metadata. Because full-length 16S rRNA gene sequencing was used, taxonomic resolution to species level was supported for a large fraction of oral taxa. Species assignments were retained where classifier confidence exceeded predefined thresholds, with ambiguous features collapsed to genus (Johnson et al., 2019; Bailén et al., 2020). The original study enrolled 127 participants, 64 HPV-positive and 63 HPV-negative, mixed sex with detailed clinical information, including HPV status based on validated genotyping methods. For the purposes of this investigation, we included all samples with complete HPV phenotype annotations and defined two comparison groups: HPV-positive versus HPV-negative individuals. Only fully anonymised FASTQ files and non-identifiable metadata were accessed for this work. No new samples were collected and no sensitive personal information was handled. Data retrieval and handling adhered strictly to FAIR principles, ensuring transparency, reproducibility, and responsible use of publicly archived human-derived sequencing datasets.

### Oral HPV status and metadata curation

Oral HPV status was obtained from the accompanying metadata provided in the original BioProject. HPV detection was performed by the primary study using validated molecular assays, and HPV status was recorded as a binary variable (HPV-positive or HPV-negative). No information on viral load, transcriptional activity, or HPV genotype stratification was available and therefore not incorporated into the present analysis. Sample-level metadata were curated to ensure consistency and completeness across microbiome and HPV datasets. Only samples with unambiguous HPV status and corresponding 16S rRNA sequencing data were retained. Demographic and clinical covariates provided in the original metadata were reviewed for completeness. Variables with substantial missingness or inconsistent annotation were excluded from multivariate modelling to avoid introducing bias. All metadata fields were harmonised and aligned with microbiome feature tables using unique sample identifiers. HPV status was treated as an exposure variable rather than a stratifying factor in unsupervised analyses to prevent circularity. This curation strategy ensured that ecological structure and virulence organisation of the oral microbiome were inferred independently of HPV status, allowing unbiased assessment of HPV-microbiome associations.

### 16S rRNA gene sequencing data processing

Raw 16S rRNA gene amplicon sequencing reads were downloaded from the NCBI and processed using a reproducible, standardised microbiome genomics pipeline. Sequencing adapters and low-quality bases were removed, and reads were quality filtered to exclude sequences below minimum length and quality thresholds. Denoising and error correction were performed to resolve amplicon sequence variants (ASVs), allowing high-resolution representation of bacterial features while minimising sequencing artefacts. Chimeric sequences were identified and removed using consensus-based methods. ASVs were taxonomically assigned against a curated reference database appropriate for oral microbiome profiling, with assignments retained at the genus level for downstream analyses to ensure robustness and comparability across samples. Samples with insufficient sequencing depth after quality control were excluded. Feature tables were constructed using ASV counts and subsequently filtered to remove low-prevalence taxa present in a minimal number of samples. This preprocessing strategy balanced retention of biologically relevant diversity with reduction of sparse, noise-driven features, enabling reliable ecological, compositional and network-based analyses.

### Taxonomic assignment and compositional data transformation

Following denoising, amplicon sequence variants were taxonomically assigned using a curated 16S rRNA reference database optimised for oral microbiome profiling. Taxonomic classification was performed using a naïve Bayes-based classifier, with confidence thresholds applied to minimise spurious assignments. To ensure robustness and reduce overinterpretation of sparse features, downstream analyses were conducted at the genus level. Because 16S rRNA sequencing data are inherently compositional, raw count tables were transformed prior to statistical analysis. Zero counts were addressed using a minimal pseudocount approach, after which centred log-ratio (CLR) transformation was applied to normalise feature distributions and enable valid multivariate and correlation-based analyses. Relative abundance estimates were retained solely for descriptive visualisation. All ordination, ecotype, network and variance-partitioning analyses were performed on CLR-transformed data to mitigate compositional bias and spurious correlations. This transformation framework ensured that ecological structure and taxa-virulence relationships reflected true relative differences in community organisation rather than artefacts of sequencing depth or library size (Callahan et al., 2016; Parks et al., 2025).

### Microbiome diversity and community structure analyses

Microbiome diversity was assessed at the sample level using standard alpha diversity metrics, including Shannon diversity and observed genus richness, calculated on rarefied feature tables to ensure comparability across samples. Differences in alpha diversity between HPV-negative and HPV-positive groups were evaluated using non-parametric statistical tests, given the non-normal distribution of diversity measures. Community structure was examined using multivariate ordination methods applied to CLR-transformed genus-level profiles. Principal component-based projections were used to visualise global patterns of microbiome variation independent of HPV status. Beta diversity and overall community dissimilarity were further evaluated using distance-based methods appropriate for compositional data. To quantify the contribution of HPV status relative to other ecological factors, variance partitioning was performed using permutational multivariate analysis of variance (PERMANOVA) with permutation-based significance testing. These analyses were designed to determine whether HPV infection independently structured the oral microbiome or whether observed associations reflected alignment with broader ecological gradients defined by diversity and functional organisation (Gloor et al., 2017).

### Ecotype identification and unsupervised clustering

To identify stable oral microbiome community states independent of HPV status, we performed unsupervised ecotype analysis on CLR-transformed genus-level profiles. Dimensionality reduction was first applied to capture dominant axes of variation while preserving ecological structure. Clustering was then performed using model-based and distance-based approaches to identify reproducible groupings of samples in microbial community space. The optimal number of ecotypes was determined using internal validation metrics, including silhouette width and cluster stability across resampling. Convex hulls were used to visualise within-ecotype coherence and overlap between ecotypes in reduced-dimensional space. Importantly, HPV status was not included as a clustering variable to avoid circular inference. Following ecotype assignment, HPV prevalence across ecotypes was assessed using contingency analyses to test whether HPV infection preferentially segregated into specific microbiome states. This strategy allowed direct evaluation of whether oral HPV defines discrete microbiome ecotypes or instead distributes across broader ecological configurations. All network inference was performed on CLR-transformed data with FDR control, reducing spurious correlations inherent to compositional counts. Primary results are reported as coordination structure (edge accumulation/rewiring) rather than isolated pairwise correlations, and robustness was checked across transformations (Peschel et al., 2021; Peterson et al., 2024).

### Inferential virulence annotation and bacterial genomics mapping

To characterise functional organisation of the oral microbiome beyond taxonomic composition, bacterial genera were annotated for inferential virulence-associated potential using curated bacterial genomics databases and genomics resources. Primary virulence annotations were derived from the Virulence Factor Database (VFDB), which provides a manually curated and hierarchically classified catalogue of experimentally validated bacterial virulence factors, grouped by functional mechanism. VFDB annotations were cross-referenced with the Victors database and the Bacterial and Viral Bioinformatics Resource Centre (BV-BRC, formerly PATRIC), which integrate peer-reviewed virulence gene curation with comparative pathogenomics. These resources were used to establish conservative, literature-supported mappings between bacterial genera and virulence domains (Yost et al., 2018; Sayers et al., 2019; Liu et al., 2022; Olson et al., 2023). Each genus was mapped to one or more biologically defined virulence domains based on documented genomic capacity for epithelial adhesion, tissue invasion, immune-interface modulation, persistence/biofilm formation, and toxin or secretion system activity. Annotations were derived from established virulence factor databases and prior functional microbiome studies, ensuring conservative and literature-supported assignments (Douglas et al., 2020; Lin et al., 2020). To contextualise virulence-associated ecological patterns, we compiled a literature-informed reference list of oral taxa previously linked to epithelial interaction, inflammation, or cancer-associated microenvironments; this curation was used solely for interpretive annotation and not for defining analytical categories or causal inference. Two composite virulence metrics were used. First, the Virulence Ecology Index (VEI) was used as the sample-level virulence-pressure metric for ecological terrain analysis. VEI summarises virulence-aligned ecological pressure derived from genera conservatively mapped to curated virulence domains. Second, the Virulence-Oncogenic Dysbiosis Score (VODS) was used as the aggregate module-level virulence burden metric for distributional and coordination analyses. VODS was defined as the unweighted sum of the five sample-level virulence module scores: VODS = Adhesion + Invasion + Immune-interface defence + Persistence/Biofilm + Toxin/Secretion damage. For ecological terrain analysis, samples were dichotomised at the median VEI and median Shannon diversity to define four quadrants (Q1-Q4). Associations between quadrant membership and HPV positivity were estimated using logistic regression with Q1 as the reference category, reporting odds ratios, 95% confidence intervals, and two-sided p-values. Because median-based quadrant definitions can be threshold-sensitive, the quadrant analysis was interpreted as an ecological visualization framework rather than as the sole basis for inference.

Virulence mapping was performed at the genus level to maintain compatibility with 16S rRNA data while capturing higher-order functional organisation shared across species and strains. Genera without clear or reproducible evidence of virulence-associated functions were excluded from virulence analyses to minimise misclassification. This bacterial genomics-informed annotation framework enabled integration of compositional data with functional potential, allowing downstream analyses to focus on how virulence programs are distributed and coordinated across microbial communities rather than inferred from isolated taxa (Escapa et al., 2018; Torralba et al., 2021). However, this study did not infer actual gene presence, gene expression, or biological activity.

### Rationale and justification for genus-level virulence inference from 16S rRNA data

We did not infer strain-specific genes or expression. We quantify VFDB-aligned virulence potential categories at genus/community level and focus inference on coordination architecture, not gene presence. This is consistent with established practice for marker-gene functional inference when framed as potential and supported by curated reference knowledge (Earl et al., 2018; Douglas et al., 2020; MatChado et al., 2024). We used VFDB’s *classification schema* to organise virulence mechanisms and assigned taxa only when supported by reproducible evidence, triangulated with Victors and BV-BRC. We excluded ambiguous taxa and interpreted results at module/category level. VFDB was explicitly designed to support pan-bacterial microbiome analyses via a unified classification (Liu et al., 2022). To minimize genus-level mapping to collapse strain-level heterogeneity, we restricted our primary endpoints to ecological state and network coordination, which are robust to taxonomic substitution under functional redundancy. We explicitly avoided strain claims and conducted sensitivity analyses removing ambiguous genera and varying annotation sets. Under the conservative genus-to-virulence mapping framework, 23 of 150 genera (15.3%) were assigned to at least one curated virulence domain, whereas 127 of 150 genera (84.7%) were not assigned because the available evidence was insufficient, ambiguous, or not reproducibly linked to the predefined domain framework. All primary virulence analyses were therefore restricted to the conservatively mapped genera, and inference was made at the level of module coordination and ecological structure rather than individual taxon pathogenicity. Functional redundancy is a well-described property of human-associated microbiomes and supports community-level functional inference (Tian et al., 2020; Wade, 2021).

### Statistical analysis

All analyses were performed using R (v4.3) and Python (v3.11) followed a rigorous, compositionality-aware statistical framework integrating diversity metrics, differential abundance testing, functional modelling and integrative score evaluation. Alpha diversity was compared using Wilcoxon tests, and beta diversity differences were assessed via PERMANOVA with dispersion checks (Anderson, 2021; Su et al., 2024; Silva et al., 2025). Differential abundance was identified using ANCOM-II, DESeq2, and LEfSe, while functional pathway differences were modelled with ALDEx2 and MaAsLin2, adjusting for relevant covariates. Non-parametric and permutation-based statistical approaches were selected to accommodate the distributional properties and compositional nature of 16S rRNA sequencing data (Weiss et al., 2016). Spearman rank correlation was used to quantify genus-virulence associations because it is robust to non-normality, monotonic but non-linear relationships, and outliers common in microbiome datasets (Weiss et al., 2016). Correlation-based coordination analysis was used to evaluate community-level genus-module organization rather than isolated taxa. Spearman correlations on CLR-transformed abundances were chosen for robustness to non-normality and compositional effects. However, such analyses remain sensitive to sparsity, indirect dependence, and threshold choice, and therefore were interpreted as ecological coordination summaries, not causal interactions. All analyses were performed on centred log-ratio-transformed abundances to mitigate spurious correlations arising from compositional constraints. Multiple testing across genus-domain associations was controlled using the Benjamini-Hochberg false discovery rate procedure. Permutation testing was employed for comparative coordination metrics (ΔAUC) to avoid parametric assumptions and to derive empirical null distributions appropriate for complex, network-derived statistics. All p-values underwent Benjamini-Hochberg FDR correction (q < 0.05), ensuring robust, reproducible identification of taxa and virulence pathways associated with HPV status.

## Results

### Age-associated ecological constraint of the oral microbiome and limited HPV-linked taxonomic shifts

**Figure 1:**
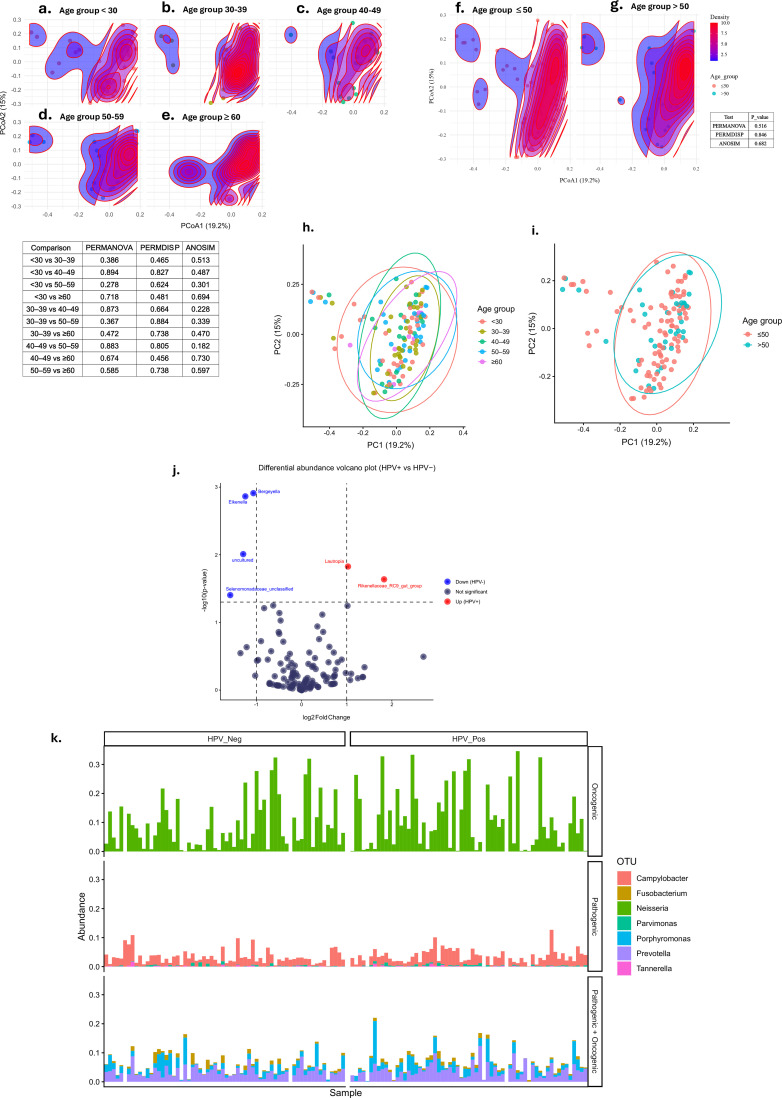
Age-associated compression of oral microbiome ecological space and modest HPV-linked taxonomic shifts **(A-E)** Across the five age strata, the oral microbiome demonstrates a gradual, monotonic compression of compositional space, transitioning from a heterogeneous, multimodal ecosystem in younger individuals to a constrained, low-variance configuration in older age. Importantly, this pattern reflects variance reduction rather than discrete compositional shifts, supporting a model of continuous ecological reorganisation with ageing. **(F, G)** The dichotomous comparison highlights a variance shift rather than a centroid shift. While both age groups occupy overlapping regions of PCoA space, the >50 group exhibits loss of ecological breadth rather than displacement to a distinct compositional state. This suggests that ageing primarily acts to constrain microbiome configuration space, rather than to induce a new dominant microbiome composition. **(H)** Across age strata, ellipses overlap substantially, indicating no discrete age-specific microbiome states. Instead, ageing is associated with a progressive reduction in dispersion (variance) rather than a systematic shift in centroid position. This pattern supports continuous ecological canalisation of the oral microbiome across adulthood. **(I)** Two groups show substantial centroid overlap, indicating no discrete age-specific microbiome states. The dominant difference lies in variance rather than position. **(J)** Volcano plot showing genus-level differential abundance comparing HPV-positive versus HPV-negative samples. Significant differential shifts were observed including enrichment of *Lautropia* and *Rikenellaceae RC9 gut group* in HPV-positive samples and depletion of genera such as *Bergeyella*, *Eikenella*, and *Selenomonadaceae* (unclassified) in HPV-positive samples. **(K)** Stacked bar plots showing the relative abundance of oral bacterial genera across individual samples, stratified by HPV-negative (HPV_Neg) and HPV-positive (HPV_Pos) groups. Samples are displayed along the x-axis, with relative abundance on the y-axis. Taxa are grouped for visualisation into three heuristic panels reflecting literature-informed context: taxa previously linked to oncogenic microenvironments (top), taxa associated with pathogenic or inflammatory states (middle), and taxa spanning both contexts (bottom). Colours indicate individual genera as shown in the legend. These groupings are provided for illustrative and interpretive purposes only and were not used to define analytical categories, derive scores, or infer causality. The figure highlights substantial inter-individual variability and overlapping abundance profiles between HPV status groups rather than discrete HPV-specific microbiome states. Differential taxa were identified using models adjusted for age as a covariate.

To establish a baseline framework for interpreting HPV-associated bacterial microbiome variation, we first examined how host age differences shape the global organisation of the oral microbiome. This is because age is a dominant determinant of microbial ecology and a potential confounder in oral HPV-microbiome associations (Kazarina et al., 2023; Yue et al., 2025). Understanding whether microbiome variation arises from discrete compositional transitions or from continuous restructuring of ecological space is essential for correctly contextualising downstream HPV-linked analyses. Across five age strata, ordination analyses revealed a little compression of oral microbiome compositional space with increasing age (**Figures 1A–E**). Younger individuals exhibited a broad, multimodal distribution, consistent with high ecological heterogeneity, whereas older age groups occupied an increasingly constrained region of ordination space.

To further test whether ageing associated with displacement toward a new dominant configuration or instead restricts ecological breadth, we performed dichotomous comparisons between younger and older individuals. These analyses demonstrated substantial overlap in centroid position (**Figures 1F, G**). Thus, ageing primarily acts to canalise the oral microbiome into a narrower range of configurations rather than inducing a qualitatively distinct community structure. Consistent with this interpretation, age-stratified confidence ellipses in a PCA plot showed extensive overlap across all strata, with a clear monotonic reduction in ellipse area rather than spatial separation (**Figure 1H**). Direct two-group comparisons similarly confirmed variance-driven differences without centroid displacement (**Figure 1I**).

Having established this age-associated ecological backdrop, we next assessed whether oral HPV infection is associated with discrete microbiome restructuring or more limited taxon-specific abundance shifts. Differential abundance analysis showed modest directional genus-level shifts, including enrichment of *Lautropia* and *Rikenellaceae RC9* gut group in HPV +ve cohort and depletion of *Bergeyella, Eikenella*, and unclassified *Selenomonadaceae in* HPV -ve cohort; however, these signals were weak and did not consistently survive multiple-testing correction across analytical frameworks (**Figure 1J**). Specifically, HPV-positive samples showed enrichment of *Lautropia* and *Rikenellaceae RC9 gut group*, alongside depletion of genera such as *Bergeyella*, *Eikenella*, and unclassified *Selenomonadaceae*. Notably, these shifts occurred within a landscape of extensive overlap, indicating that HPV infection is not associated with a wholesale reorganisation of community composition.

To visualise these taxon-level patterns in a sample-resolved manner, we examined relative abundance profiles of selected virulence-associated genera stratified by HPV status (**Figure 1K**). We observed a pronounced inter-individual variability within both HPV-positive and HPV-negative groups and substantial overlap in abundance profiles across curated taxon groupings.

To mitigate confounding by age, differential abundance analyses were additionally fitted using multivariable models with age included as a covariate; taxa reported as significant were required to retain directionality and statistical support after age adjustment.

### HPV-associated restructuring of the oral microbiome reveals and the dysbiotic ecological state

We reframed ecological virulence potential, reflecting community-level traits that favour immune evasion and chronic colonisation rather than direct suppression of host immunity (Dobson, 2025; Silva and King, 2025). We first examined HPV-associated differences in microbial composition, diversity and directional enrichment to establish whether HPV positivity corresponds to a coherent ecological state rather than stochastic taxon-level variation (Zhang et al., 2022; Dahlstrom et al., 2023).

**Figure 2:**
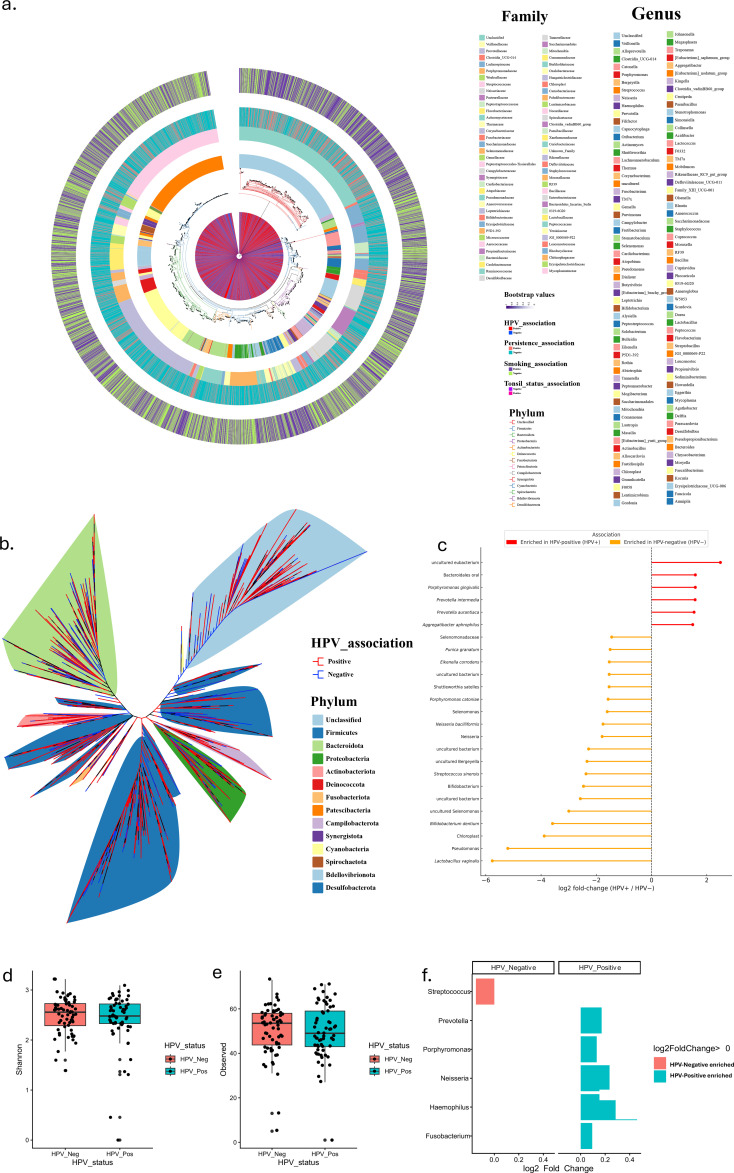
HPV-associated compositional and ecological restructuring of the oral microbiome. **(A)** Circular phylogenetic mapping of the oral microbiome showed non-random, clade-level organisation of microbial associations with oral HPV status and HPV persistence. Smoking and tonsil status exhibit broad, coherent associations across multiple clades, highlighting these host factors as dominant axes shaping oral microbiome structure and potential modifiers of HPV-microbiome relationships. **(B)** Radial phylogenetic mapping demonstrates that bacterial associations with oral HPV are widely distributed across multiple phyla rather than confined to a single lineage. Both positive and negative HPV associations occur within major phyla, with pronounced within-phylum heterogeneity. **(C)** Differentially enriched oral taxa by HPV status showing genera with the strongest differential abundance between HPV-positive and HPV-negative samples, expressed as log2 fold change (HPV+/HPV−), with the dashed vertical line indicating no difference. HPV-positive samples are selectively enriched for classical periodontal and anaerobic taxa, including *Porphyromonas gingivalis*, *Prevotella intermedia*, *Prevotella aurantiaca*, *Aggregatibacter aphrophilus* and *Bacteroidales* lineages, consistent with an invasive, proteolytic and inflammation-associated oral microbiome. **(D, E)** Within-sample microbial diversity between HPV-negative and HPV-positive groups using Shannon diversity and observed taxa richness, HPV-positive samples showed a modest but consistent shift toward higher alpha diversity, reflected by elevated Shannon indices and increased observed richness, indicating a more taxonomically complex oral microbial community although statistically it was not significant. **(F)** The direction of differential abundance stratified by HPV status, where bars indicate the number of taxa with positive or negative log2 fold change. The HPV-positive group is dominated by taxa with log2FoldChange > 0, suggesting a strong skew toward microbial enrichment in HPV-positive samples. **(C)** displays the complete set of discriminative features with effect sizes, whereas **(F)** summarizes a curated subset of representative genera to provide an interpretable overview of the dominant HPV-associated microbial shifts. Discrepancies between **(C, F)** reflect intentional rank harmonization (species-to-genus collapsing) and feature curation for interpretability. Both panels derive from the same Linear Discriminant Analysis Effect Size analysis and are directionally concordant at the genus level. Differentially enriched oral taxa by HPV status are shown as effect-size directions rather than definitive FDR-significant hits. These patterns are presented as descriptive compositional shifts.

To resolve whether HPV-associated microbiome differences reflect isolated taxa or coordinated evolutionary patterns, we applied phylogeny-aware analyses that map microbial associations onto evolutionary structure. This descriptive approach summarized whether HPV alignment was randomly distributed or conserved across clades, providing mechanistic insight into how host-virus interactions reorganise the oral microbiome (**Figures 2A, B**). Differential abundance analysis showed a marked polarisation of the oral microbiome by HPV status (**Figure 2C**). HPV-positive samples were selectively enriched for periodontal and anaerobic taxa with established roles in tissue invasion, proteolysis and inflammation, including *Porphyromonas gingivalis*, *Prevotella intermedia*, *Prevotella aurantiaca*, *Aggregatibacter aphrophilus* and multiple Bacteroidales lineages. In contrast, HPV-negative samples were dominated by commensal and health-associated taxa such as *Lactobacillus vaginalis*, *Bifidobacterium* spp., *Streptococcus sinensis*, *Neisseria* spp. and *Eikenella corrodens*, suggesting a compositionally distinct and less pathogenic microbial ecosystem. The majority of differentially abundant taxa exhibited positive log2 fold changes in HPV-positive samples, whereas HPV-negative samples showed few enriched taxa. This imbalance suggests an overall expansion of the microbial niche space in HPV positivity, consistent with ecological release or reduced host constraint (Aggarwal et al., 2023; Chung et al., 2024). At the community level, samples showed no statistical significant difference within-sample diversity (Shannon diversity) (**Figures 2D, E**).

The parallel increase in both richness and evenness suggest that HPV positivity was associated with a broader and more permissive microbial landscape rather than dominance by a single taxonomic group. Directional analysis of differential abundance further emphasized this asymmetry (**Figure 2F**).

### HPV status did not define oral microbiome ecotypes

**Figure 3:**
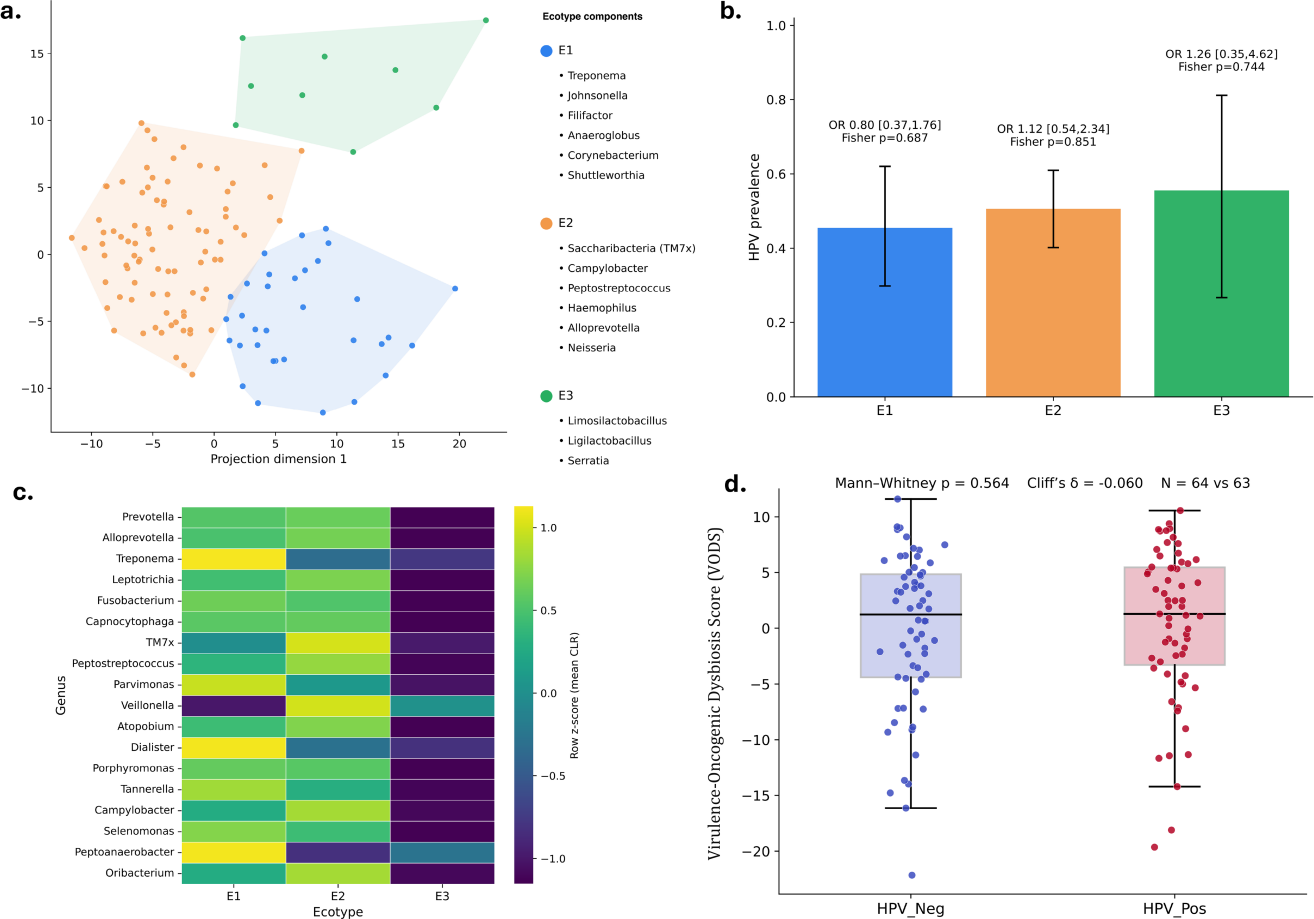
Oral microbiome ecotypes and their relationship to HPV status. **(A)** Unsupervised projection of CLR-transformed genus-level oral microbiome profiles identifies three distinct ecotypes occupying separable ecological space. Ecotypes were derived independently of HPV status. **(B)** HPV prevalence across ecotypes shows no strong enrichment or depletion in any single ecotype. **(C)** Each ecotype is defined by a distinct, non-overlapping set of discriminating genera identified using sparse multinomial modelling. Row z-scored mean CLR values reveal coordinated, ecotype-specific taxonomic signatures. **(D)** Comparison of the VODS between HPV-negative and HPV-positive individuals shows substantial overlap and no significant difference.

Whether oral HPV infection imposes a dominant restructuring of the oral microbiome remains unresolved, with prior studies variably reporting enrichment of individual taxa but rarely testing community-level organisation (Yost et al., 2018; Zhang et al., 2022; Escobar Marcillo et al., 2025). Our central hypothesis was that, if HPV acts as a major ecological driver, then HPV-positive samples would segregate into discrete microbiome states characterised by coherent taxonomic and functional signatures (Lin et al., 2020; Dahlstrom et al., 2023). To test this directly, we first defined unsupervised oral microbiome ecotypes independent of HPV status and then systematically examined how HPV prevalence, taxonomic structure and an aggregate oncogenic-virulence dysbiosis score aligned with these ecotypes (**Figure 3**). Projection of cantered log-ratio (CLR)-transformed genus-level profiles revealed three reproducible oral microbiome ecotypes, each occupying a distinct region of ecological space (**Figure 3A**). Importantly, HPV-positive and HPV-negative samples were interspersed across all ecotypes, suggesting that HPV infection alone does not impose a dominant community-level partitioning of the oral microbiome.

Consistent with the projection analysis, HPV prevalence did not differ substantially across the three ecotypes (**Figure 3B**). Odds ratios comparing each ecotype against the remainder of the cohort were close to 1, with confidence intervals spanning 1 and Fisher’s exact tests showed no significant enrichment or depletion of HPV positivity within any single ecotype. These results suggest that the ecotype structure observed in **Figure 3A** is not trivially explained by HPV status, effectively ruling out HPV-driven clustering as a confounder in downstream analyses. Despite similar HPV prevalence, each ecotype was characterised by a distinct set of discriminating genera (**Figure 3C**). Using a sparse multinomial modelling approach, we identified non-overlapping taxonomic signatures that differentiated the ecotypes, including enrichment of anaerobic and periodontitis-associated genera in one ecotype and distinct commensal-dominated profiles in others. Row z-scoring of mean CLR values highlighted coordinated, ecotype-specific shifts rather than isolated taxon-level differences, emphasizing that these ecotypes represent biologically meaningful community states.

Finally, we asked whether HPV infection was associated with a global shift in oncogenic-virulence potential across the oral microbiome. Comparison of the VODS between HPV-negative and HPV-positive individuals showed substantial overlap between groups, with no statistically significant difference (Mann-Whitney p = 0.56; Cliff’s δ = -0.06; **Figure 3D**).

### HPV aligns with a virulence-pressured, low-resilience oral microbiome ecological landscape

**Figure 4:**
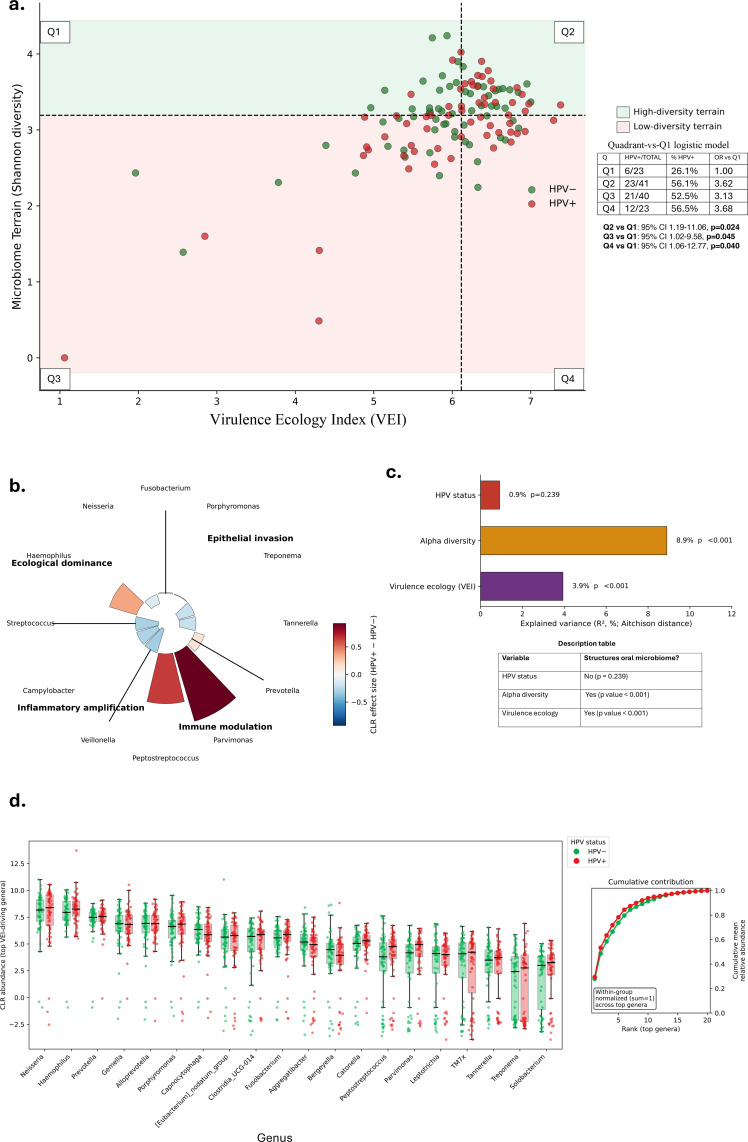
HPV-associated ecological reprogramming of the oral microbiome virulence architecture. **(A)** Individual oral sample positioned within a two-dimensional ecological space defined by oncogenic-virulence pressure (x-axis; Virulence Ecology Index, VEI) and microbiome resilience (y-axis; Shannon diversity). VEI summarises the relative enrichment of bacterial genera with documented epithelial invasion, immune modulation, and pro-inflammatory virulence programs, while Shannon diversity reflects community complexity and ecological buffering capacity. Median values of VEI and Shannon diversity (dashed lines) partition samples into four ecological states: Q1 (high diversity/low pressure; protected terrain), Q2 (high diversity/high pressure; buffered exposure), Q3 (low diversity/low pressure; latent vulnerability), and Q4 (low diversity/high pressure; danger zone). HPV-positive samples were enriched outside the protected terrain, with increased odds of HPV positivity observed in states characterised by either elevated virulence pressure, reduced ecological resilience, or both, and the highest odds in the low-diversity/high-pressure danger zone (Q4). **(B)** Depicting coordinated shifts in microbial virulence ecology associated with HPV infection. Each bar represents a bacterial genus with established links to epithelial invasion, immune modulation, inflammatory amplification, or ecological dominance. Bar height corresponds to the CLR-transformed effect size (mean HPV+ minus mean HPV−), with colour encoding the direction and magnitude of enrichment (red, higher in HPV-positive samples; blue, higher in HPV-negative samples). Genera were grouped into biologically curated virulence domains, delineated by radial separators and labelled on the outer ring, highlighting higher-order functional organisation beyond individual taxa. This architecture suggested that HPV infection was associated with structured, domain-level enrichment of virulence-linked microbial programs rather than isolated taxonomic changes. **(C)** The PERMANOVA analysis identified virulence ecology and diversity as independent structuring axes of the oral microbiome. Alpha diversity explains the largest independent fraction of variance (8.9%, p < 0.001), followed by the Virulence Ecology Index (VEI; 3.9%, p < 0.001), whereas HPV status alone accounts for a small and non-significant component (0.9%, p = 0.239). P-values were estimated by permutation testing (999 permutations). **(D)** Multiple paired box plots showed centred log-ratio (CLR)-transformed abundances of the top 20 VEI-driving genera, ordered by overall contribution to the virulence ecology. Each genus is displayed as paired distributions comparing HPV-negative and HPV-positive samples, illustrating a consistent directional shift in abundance profiles rather than dominance by a single taxon. The cumulative mean relative abundance across the ranked genera, calculated within each HPV group and normalised to sum to one, highlighted how virulence-associated biomass was distributed across taxa in HPV-negative versus HPV-positive microbiomes. (i) Several HPV-positive points in Q1 and other quadrants are touched by the median lines, therefore, visual inspection may undercount or overcount these boundary samples compared with their numerical quadrant assignment in the analysed dataset. (ii) Odds ratios were estimated using logistic regression with Q1 as the reference category; 95% confidence intervals and two-sided Wald p-values are reported (iii) In Figure **(C)**, PERMANOVA analysis, the bars are not compared to each other, they are all components of a multivariate model.

While taxonomic enrichment analyses establish that HPV positivity associates with a dysbiotic oral microbiome, they do not resolve whether HPV aligns with specific ecological configurations defined by coordinated virulence pressure and community resilience (Feng et al., 2024). The word “aligns with” means a statistical and ecological co-occurrence framework rather than as evidence of direct causation or mechanistic viral control of microbiome function. To address this gap, we integrated diversity metrics, virulence-linked taxonomic structure and multivariate community analyses to test whether HPV persistence maps onto distinct regions of a microbiome ecological landscape rather than acting as an isolated driver of compositional change. Mapping individual samples onto a two-dimensional ecological terrain revealed that HPV positivity is non-randomly distributed across microbiome states defined by virulence pressure and resilience (**Figure 4A**). HPV-positive samples were depleted from the protected terrain characterised by high diversity and low virulence pressure (Q1) and instead accumulated in ecological states marked by either elevated virulence pressure, reduced diversity, or both (Q2,Q3,Q4, all with OR above 3). The highest odds of HPV positivity were observed in the low terrain, high-pressure danger zone (Q4), suggesting that in this cohort, HPV infection aligns with convergent ecological vulnerabilities rather than with virulence enrichment or diversity loss alone (**Figure 4A**).

At a higher organisational level, HPV infection was associated with a structured reconfiguration of virulence-linked microbial programs (**Figure 4B**). Rather than isolated taxon-specific effects, HPV-positive samples exhibited coordinated enrichment across biologically coherent virulence domains encompassing immune modulation. This domain-level architecture supports a model in which HPV aligns with a pathogenic-oncogenic microbiome ecology that is functionally organised, reinforcing the ecological signal observed in the terrain analysis (Lin et al., 2020; Byrd et al., 2025).

Multivariate modelling further clarified the relationship between HPV status, virulence ecology and global community structure (**Figure 4C**). We wanted to know how much of the total microbiome community variation was explained by this variable, after controlling for the others? To address this question we used PERMANOVA analysis similar to published studies in microbiomes analysis (Anderson, 2021; Su et al., 2024; Silva et al., 2025). This analysis showed that alpha diversity and virulence ecology independently explained substantial proportions of microbiome variance, whereas HPV status alone accounted for a small and non-significant fraction (R^2^ 8.9% and 3.9% respectively, both p-values <0.001), similar to previous findings (Zhang et al., 2022; Maidment et al., 2025).

Finally, multiple examination of the top virulence ecology-driving genera showed a consistent, directional skew in abundance profiles in HPV-positive samples without dominance by any single taxon (**Figure 4D**). The cumulative biomass analysis revealed that virulence-associated signal in HPV-positive microbiomes was distributed across multiple genera, reinforcing the concept of diffuse ecological restructuring. Although no individual genus reached statistical significance after multiple-testing correction, biologically, the concordant shifts across taxa emphasize that oral HPV-associated dysbiosis is an emergent, system-level property rather than a consequence of discrete microbial overgrowth (Foster et al., 2017; Gilbert and Lynch, 2019).

### HPV-associated rewiring of virulence coordination networks in the oral microbiome

While compositional and diversity analyses establish that HPV positivity aligns with a dysbiotic oral microbiome, they do not resolve how virulence functions are ecologically organised and coordinated across taxa (Radaic and Kapila, 2021; Chan et al., 2022; Escobar Marcillo et al., 2025). Because pathogenicity often emerges from coordinated interactions between microbes and functional programs rather than from isolated abundance shifts, we next interrogated the network structure of taxa-virulence relationships to determine whether HPV infection is associated with systematic rewiring of virulence ecology rather than uniform amplification of virulence burden (Torralba et al., 2021).

**Figure 5:**
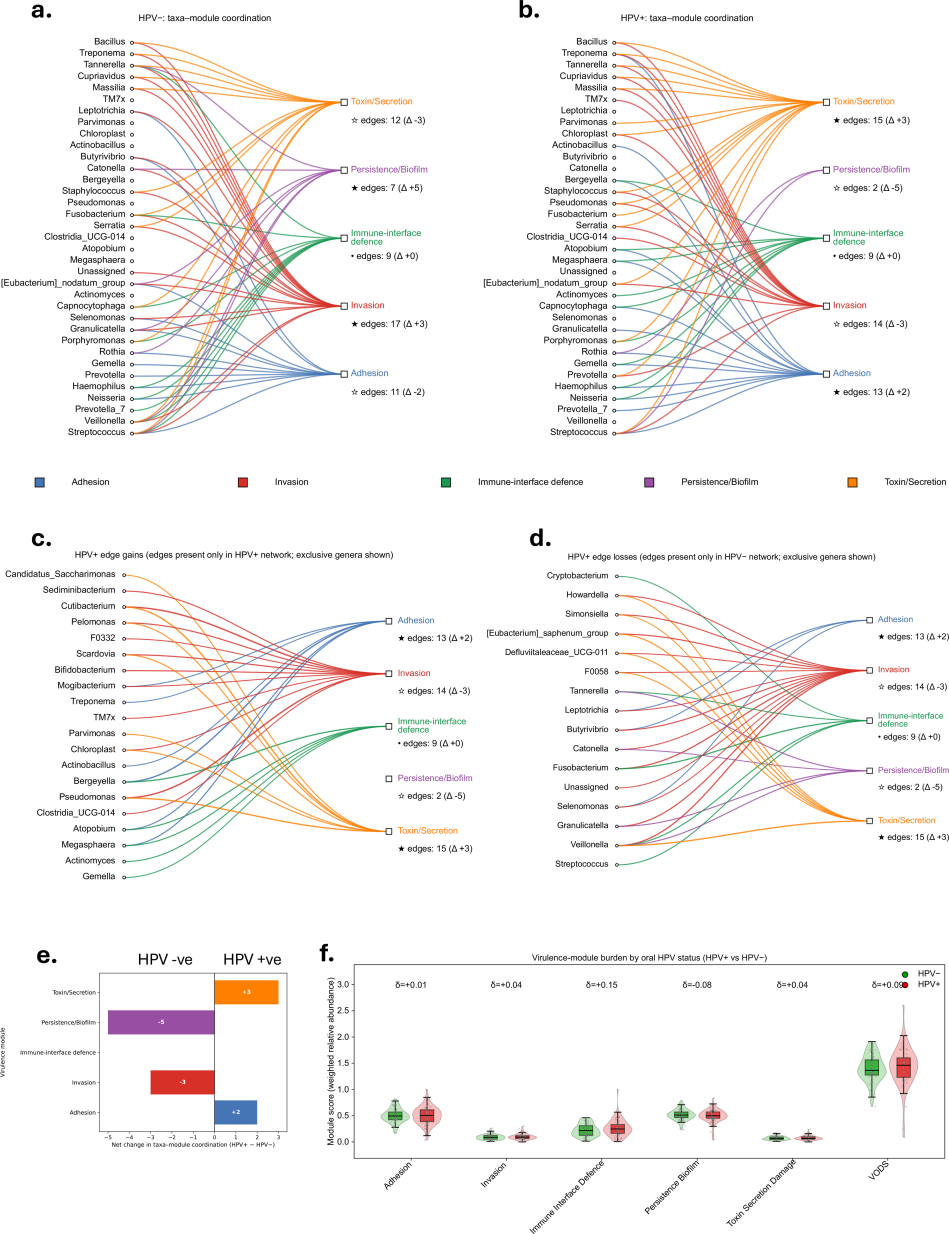
Structured network-level reprogramming of taxa and genera virulence ecology by HPV infection. **(A, B)** HPV-associated structured virulence ecology by taxa-module coordination networks. Bipartite networks depict coordinated associations between oral bacterial taxa (left node) and virulence-linked functional modules (right nodes) stratified by HPV status (left panel, HPV−, right panel, HPV+). Edges represent significant positive correlations between abundance and module scores (Spearman correlation, FDR-adjusted), with edge thickness proportional to association strength and colour indicating the virulence module involved. Comparison across panels reveals almost similar HPV-associated rewiring of the virulence ecology. The values in parentheses indicate the difference (Δ) relative to the opposite HPV status. **(C, D)**. Network panels depicting directional rewiring of genus-virulence interactions associated with HPV status. Left (HPV+ edge gains) shows genus-module connections present exclusively in HPV-positive samples, whereas right (HPV+ edge losses) shows connections present exclusively in HPV-negative samples. Nodes on the left represent bacterial genera, nodes on the right represent virulence-linked functional modules (adhesion, invasion, immune-interface defence, persistence/biofilm, toxin/secretion). Edges denote significant positive genus-module coordination (Spearman correlation, FDR-adjusted). These panels illustrate structured ecological rewiring of virulence coordination with HPV infection rather than uniform changes in overall virulence burden. **(E)** Net rewiring of taxa-virulence module coordination associated with HPV status, horizontal bars summarise the net change in significant genus-virulence module associations between HPV-positive and HPV-negative samples, calculated as HPV+ minus HPV−. Positive values indicate modules with a net gain of coordinated taxa-module edges in HPV-positive samples, whereas negative values indicate net losses relative to HPV-negative samples. Numeric values shown within bars denote the magnitude and direction of net change, providing an integrated, module-level view of HPV-associated ecological rewiring across virulence functions. **(F)** No individual virulence module demonstrated a statistically significant difference in mean abundance between HPV-positive and HPV-negative individuals, suggesting that HPV-associated effects are not driven by simple increases in virulence burden. This motivated downstream analyses focusing on ecological coordination and network structure. In Figure **(F)**, only effect sizes (Cliff’s δ) are shown above each module to convey magnitude and direction. Positive indicates higher in HPV positive samples. The bold star symbol denotes the direction of enrichment. ★ indicating a net gain of coordinated edges in that HPV group. ☆ indicating a net loss • indicating no net difference.

Our analysis HPV-taxa modulation showed almost similar patterns for HPV-associated restructuring of genus-module coordination (**Figures 5A, B**). We observed an almost similar qualitative shift in the type of virulence ecology present rather than a global increase in virulence. Module-level edge annotations highlighted asymmetric gains and losses across functional domains, demonstrating that HPV infection does not reconfigure virulence coordination patterns. Directional rewiring analysis further clarified the nature of this ecological analysis for edge gains at genera level (**Figures 5C, D**). HPV-positive-specific edge gains networks showed the emergence of genus-module connections that were absent in HPV-negative samples, whereas reciprocal panels highlighted connections lost with HPV positivity. These gains and losses were non-random and module-specific, reinforcing that HPV-associated effects reflect structured ecological rewiring at the genus level rather than stochastic edge turnover. Notably, no single genus dominated these changes, supporting a distributed, system-level reorganisation of virulence coordination.

Integration of these effects at the module level revealed clear net gains and losses of coordinated associations across virulence functions (**Figure 5E**). Certain modules exhibited a net increase in coordinated taxa-module edges in HPV-positive samples, whereas others showed net losses, providing a concise summary of how HPV reshapes the balance of virulence strategies within the oral microbiome. This module-level perspective reinforced that HPV-associated dysbiosis operates through redistribution of functional coordination rather than uniform expansion.

Importantly, direct comparison of mean virulence module abundances showed no statistically significant differences between HPV-positive and HPV-negative groups (**Figure 5F**). This absence of abundance-level effects suggests that HPV-associated microbiome alterations are not driven by increased virulence load per se, but instead arise from changes in how virulence functions are ecologically coordinated across genera. However, these network findings were interpreted as summaries of ecological coordination across taxa and inferred virulence modules, rather than as evidence of direct microbial interaction. Because correlation-based networks in compositional microbiome data are influenced by sparsity, indirect dependence, and transformation choices, they cannot establish causal, physical, or mechanistic relationships between organisms or functions.

### HPV-associated reorganisation of virulence ecology through coordinated taxa-module interactions

We analysed whether HPV infection aligns with an increase in virulence burden per se, or with a deeper reorganisation of how virulence programs are distributed and coordinated across the community. To distinguish marginal effects from ecological structure, we interrogated;

- distributional shifts in module scores and contributions.
- network-level coordination between taxa and virulence modules, and.
- how this coordination reorganises along the composite virulence-oncogenic dysbiosis axis (VODS).

**Figure 6:**
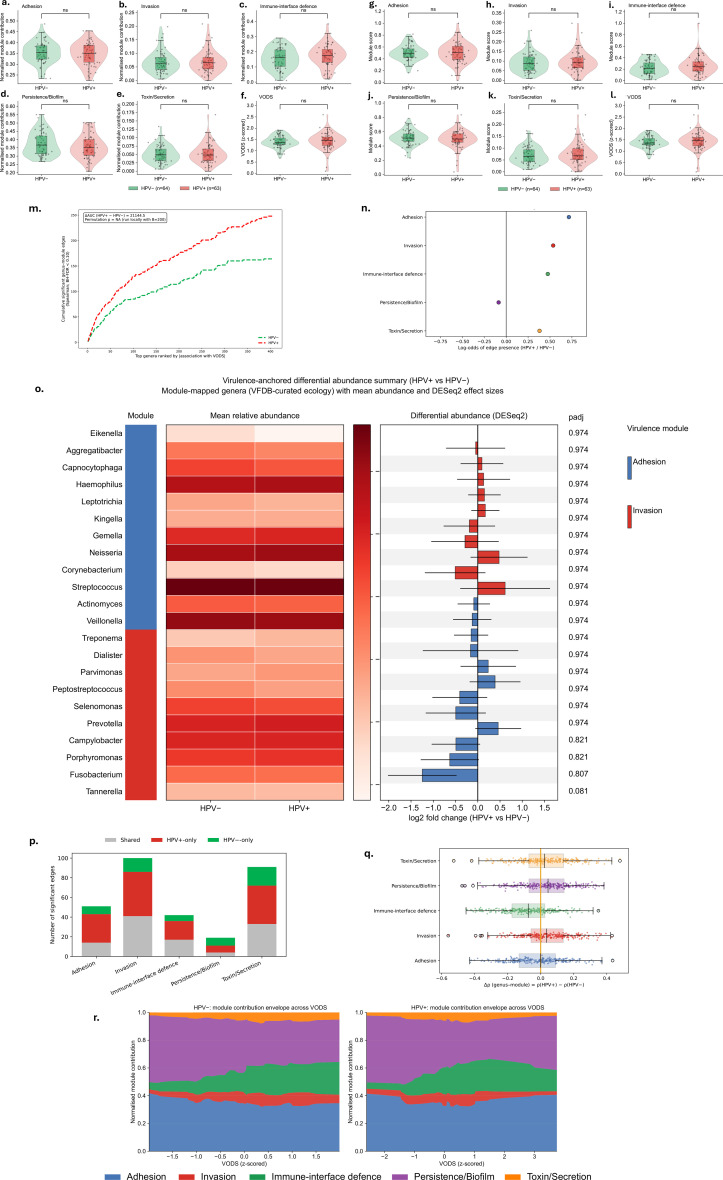
Structured redistribution and coordination of virulence programs across the oral microbiome by HPV status. **(A–F)** Raincloud plots show the full distribution of normalised virulence module contributions and **(G–L)** show raw virulence module scores across HPV− and HPV+ samples, alongside the composite Virulence-Oncogenic Dysbiosis Score (VODS). Across all modules, HPV+ and HPV− groups exhibit substantial overlap in central tendency, with no individual module demonstrating a statistically significant shift after multiple-testing correction. Importantly, the dot-based representation reveals broad within-group heterogeneity and continuous variation rather than discrete module-specific separation by HPV status. **(M)** Cumulative accumulation curves show the number of significant genus-virulence module coordination edges (Spearman correlation, Benjamini-Hochberg FDR < 0.10) as oral bacterial genera are ranked by the absolute strength of their association with the Virulence-Oncogenic Dysbiosis Score (VODS). The x-axis orders genera from lowest to highest association with VODS, while the y-axis represents the cumulative count of significant genus-module edges detected within each HPV group. HPV-positive samples accumulate coordinated virulence edges more rapidly and to a higher total than HPV-negative samples, suggesting a greater overall density of structured virulence coordination among VODS-aligned taxa. Statistical comparison of the two accumulation profiles is summarised by the difference in cumulative area (ΔAUC) and an empirical bootstrap p-value (B = 200), indicating no statistically significant global separation between curves. With a long x-axis and cumulative y-values approaching ~150 edges, absolute AUC values naturally become large. Interpretation should therefore focus on the direction of ΔAUC and its resampling-based p-value, rather than the absolute magnitude of the area difference. **(N)** Dot plot showing the log-odds ratio of significant genus-module coordination edges in HPV-positive relative to HPV-negative samples (FDR < 0.10), with the vertical line indicating log-odds = 0. HPV positivity is associated with a pronounced enrichment of coordination edges involving adhesion, invasion and immune-interface defence modules and toxin/secretion suggesting preferential coupling of taxa with epithelial attachment, tissue penetration, and host-microbe interface functions. **(O)** Genera were mapped to VFDB-curated virulence ecology modules and ordered by module blocks, summarising both compositional and differential signals. The heatmap shows mean relative abundance (log10-scaled) in HPV- and HPV+ samples, while horizontal bars depict DESeq2 log2 fold changes (HPV+ vs HPV-) with 95% confidence intervals; adjusted p-values are reported at right. Differential signals are concentrated within adhesion and invasion modules, indicating selective enrichment and depletion of taxa linked to epithelial attachment and tissue invasion in HPV-positive samples. **(P)** Stacked bar plots show the number of significant genus-module coordination edges (Spearman correlation, FDR < 0.10) stratified into edges shared between HPV-positive and HPV-negative samples and edges specific to both HPV-positive and HPV-negative samples. HPV-positive samples display a marked excess of HPV-specific rewiring within adhesion, invasion, immune-interface defence and toxin/secretion modules, indicating extensive restructuring of host-interactive and invasive virulence programs. **(Q)** The Δρ = ρ(HPV+) − ρ(HPV−), where ρ denotes Spearman correlation between genus relative abundance and module score. Each point represents a single genus-module edge, and boxes summarise the median and interquartile range, the vertical reference line at Δρ = 0 indicates no HPV-associated shift. Invasion, toxin/secretion and persistence/biofilm modules exhibit systematic rightward shifts, indicating stronger genus-module coupling in HPV-positive samples. **(R)** Show the normalised contribution of the five virulence modules across the Virulence-Oncogenic Dysbiosis Score (VODS) for HPV−(left) and HPV+ (right) samples. In both samples, persistence/biofilm and adhesion functions dominate across the gradient with a gradual increase in immune-interface defence at higher VODS, consistent with a stabilised but weakly invasive ecological state. (i) In figure **(M)**, the ΔAUC value is large because the area is computed over cumulative edge counts across approximately 150 ranked genera, the units are effectively “edges × rank” (an unnormalized area). (ii) These accumulation curves reflect ordered aggregation across genera ranked by VODS association and do not imply temporal or longitudinal dynamics. (iii) FDR refers to the Benjamini-Hochberg False Discovery Rate Adjusted p-values are displayed to show that no individual genus-level DESeq2 feature reached the prespecified FDR threshold; the figure is therefore interpreted as a virulence-anchored effect-size summary rather than as evidence for single-genus significance.

We first asked whether HPV positivity is accompanied by simple shifts in virulence module abundance or weighting. Across both normalised module contributions and raw module scores, HPV-positive and HPV-negative samples showed broad overlap and substantial within-group heterogeneity, with no module exhibiting a robust shift after multiple-testing correction (**Figures 6A–L**). This distributional continuity argues against a model in which HPV positivity is explained by uniform amplification of any single virulence program, motivating analyses focused on coordination and organisation rather than mean differences.

To test whether HPV infection instead associates with structured coordination among virulence-aligned taxa, we quantified the accumulation of significant genus-module correlations as genera were ranked by their alignment with VODS. HPV-positive samples accumulated coordinated edges more rapidly and reached a higher total edge count than HPV-negative samples, suggesting denser virulence coordination concentrated among VODS-associated taxa (**Figure 6M**). Although the global separation of curves did not reach statistical significance, the directional pattern supports HPV-linked enrichment of coordinated virulence structure rather than diffuse, unstructured variability.

We then decomposed this coordination signal by module to identify functional biases. HPV positivity preferentially enriched coordination edges involving adhesion, invasion and immune-interface defence, with a smaller enrichment in toxin/secretion and minimal bias for persistence/biofilm (**Figure 6N**). This pattern suggests that HPV-associated restructuring disproportionately involves modules most relevant to epithelial engagement and interface modification, rather than long-term colonisation strategies.

Anchoring differential abundance (DESeq2) to virulence ecology provided an orthogonal view linking compositional shifts to functional domains. When DESeq2 effect sizes were anchored to VFDB-curated virulence modules, directional shifts were visually concentrated within adhesion and invasion blocks; however, no individual genus-level feature met the prespecified FDR threshold, indicating that the functional interpretation rests primarily on coordinated module-level structure rather than single-genus differential abundance (**Figure 6O**).

We next resolved whether HPV-linked differences reflect shared coordination patterns versus true rewiring. Partitioning of significant genus-module edges showed a marked excess of HPV-specific edges within adhesion, invasion, immune-interface defence and toxin/secretion, whereas persistence/biofilm was comparatively balanced or HPV-negative skewed (**Figure 6P**). Consistent with this, edge-strength distributions demonstrated systematic rightward shifts in change of ρ for invasion, persistence/biofilm and toxin/secretion, indicating stronger genus-module coupling in HPV-positive samples, while adhesion remained centred near zero (**Figure 6Q**), similar to existing literature on reinforcing the concept of structured ecological rewiring (Chung et al., 2024; Kamassa et al., 2025).

Finally, we examined how module composition changes along the VODS gradient within each HPV stratum to determine whether HPV modifies the internal organisation of virulence ecology across disease-relevant states. In both HPV-negative and HPV-positive samples, persistence/biofilm and adhesion contributions dominated across the VODS continuum with only gradual increases in immune-interface defence at higher VODS, consistent with a stabilised but weakly invasive configuration (**Figure 6R**).

## Discussion

This is the first study to analyse 16S rRNA oral bacterial microbiome sequencing data for virulence ecology. Our study moves beyond descriptive microbiome taxa to systems-level bacterial genomics. HPV infection reorganizes within the oral bacterial microbiome virulence ecology network without simply increasing microbiome loads. Virulence ecology emerges from community structure and function allocation, not just from which taxa dominate. The community may have “stable states” of virulence function distribution, showing resilience or redundancy, so different HPV-microbiome compositions can still produce similar virulence profiles. We showed that oral HPV infection does not impose discrete taxonomic shifts or dominant microbiome states. Instead, HPV positivity aligns with a structured, pre-existing virulence ecology within the oral microbiome context. HPV-positive samples displayed distributed enrichment of periodontal and anaerobic taxa that increased in the virulence ecological state that may favour viral persistence and oncogenic coordination. Our findings suggest that oral HPV persistence may not be driven by increased microbiome virulence load, but by how microbiome virulence programs particularly involving adhesion, invasion and immune-interface functions, are ecologically organised across oral bacterial communities. Oral HPV infection is a context-dependent ecological phenomenon that aligns with coordinated microbial virulence ecology architectures rather than reshaping microbiome composition. These findings should be interpreted within the context of asymptomatic oral HPV positivity in a non-cancer cohort and should not be conflated with HPV-driven tumour ecology. The ecological pressures operating in oral HPV carriage differ fundamentally from those in hypoxic, necrotic, immune-remodelled tumour microenvironments.

Our novel ecological bacterial genomics perspective provides a new conceptual framework with a direct relevance to oral HPV infection persistence, oncogenic risk and translational microbiome research. The translational potential of this study relies on reclassifying oral HPV infection risk based on microbiome virulence ecology rather than individual taxa. By identifying virulence ecology coordination and low-resilience ecological states that preferentially align with oral HPV persistence, it enables development of microbiome-based risk stratification biomarkers (Buytaers et al., 2024; Al-Marzooq and Christidis, 2025; Eslami et al., 2025). However, our findings are ecological and hypothesis-generating, do not directly justify immediate clinical interpretation, risk stratification, or intervention, and require validation in longitudinal, clinically annotated, multi-cohort studies with outcome data.

Zhang et al. (2022) reported significant beta-diversity separation between HPV-positive and HPV-negative individuals at a taxa level, alongside enrichment of anaerobic and periodontal taxa implicated in inflammation and tissue interface disruption but not at an ecological virulence variation (Zhang et al., 2022). Similarly, Feng et al. (2024) observed weak and inconsistent associations between oral HPV and alpha diversity (Feng et al., 2024). From a bacterial genomics standpoint, these findings collectively suggest that oral HPV does not define a discrete microbiome state, but rather associates with broader ecological virulence gradients shaped by host immunity, epithelial integrity and microbial functional organisation. The recurrent lack of stable taxonomic signatures across cohorts implies that persistence is unlikely to be driven by single organisms or simple abundance shifts. The apparent discrepancy between compositional trend plots and the DESeq2 summary reflects differences in purpose: some panels were designed to visualise directional ecological shifts, whereas the DESeq2 table applies strict multiplicity correction at the individual-feature level. Accordingly, our biological inference does not rest on any single genus-level hit. HPV appears to sample from pre-existing ecological contexts in which microbial communities are permissive to long-term epithelial colonisation. Our work builds directly on this foundation by moving beyond taxonomic comparisons to interrogate ecological virulence coordination and ecological resilience, providing a mechanistic explanation for why prior studies consistently detect association without convergence on specific bacterial taxa.

We also observed distributed enrichment of periodontal and anaerobic taxa with known roles in proteolysis, inflammation and epithelial interface modulation, alongside substantial overlap between HPV-positive and HPV-negative samples and the absence of discrete HPV-defined microbiome states. This pattern was expected because HPV establishes infection at the basal epithelial layer and persists through immune evasion and limited cytopathic effect. By explicitly modelling ecological virulence structure, our analyses emphasized that HPV status contributes little independent variance to overall microbiome composition once microbial resilience and virulence organisation are accounted for. Diversity metrics and virulence ecology emerge as the dominant structuring axes of the oral microbiome. This finding indicates that HPV does not function as a primary architect of the oral bacteriome. Rather, HPV aligns with pre-existing ecological states characterised by reduced resilience and coordinated expression of host-interactive virulence programs (Groves et al., 2021; Xu et al., 2024). These states reflect community-level organisation and functional association across taxa rather than expansion of individual organisms. This framework explains why taxonomic signatures of oral HPV positivity vary across cohorts while the biological association remains reproducible. HPV persistence may be favoured in permissive ecological contexts that are shaped upstream by host, behavioural and microbial factors and HPV merely occupies these niches rather than creating them *de novo* (Lin et al., 2020; Dickey et al., 2021; Dickey et al., 2025). The absence of a significant alpha-diversity difference is ecologically informative. It argues against wholesale collapse or expansion of community richness and instead suggests that oral HPV positivity may align with selective reorganization of host-interactive or keystone ecological networks within an otherwise overlapping diversity landscape, where coordination structure matters more than overall richness. Our ecological bacterial genomics perspective gives new insights on oral HPV infection and its persistence could be a systems-level phenomenon governed by microbiome ecological virulence community organisation.

While our primary framework emphasises community-level virulence ecology rather than single-taxon effects, several differentially abundant genera enriched in HPV-positive samples provide biologically plausible anchors to the ecological patterns observed. In particular, enrichment of periodontal and anaerobic genera including *Porphyromonas*, *Prevotella*, *Fusobacterium*, *Haemophilus* and *Neisseria* aligns with prior reports linking oral HPV positivity to taxa implicated in epithelial barrier disruption, proteolysis, immune modulation and chronic mucosal inflammation (Zhang et al., 2022; Feng et al., 2024). *Porphyromonas gingivalis* and *Prevotella* spp. are established modulators of epithelial integrity and innate immune signalling, capable of promoting a permissive inflammatory microenvironment for viral persistence, while *Fusobacterium* spp. have been implicated in mucosal immune evasion and oncogenic co-factors in head and neck epithelia (Kwak et al., 2024). Conversely, taxa depleted in HPV-positive individuals, including commensal-associated genera such as *Streptococcus* and *Rothia*, are consistent with ecological states characterised by reduced colonisation resistance and diminished barrier-protective functions (Escobar Marcillo et al., 2025). Importantly, we interpret these associations at the level of ecological function rather than pathogenic identity, recognising that genera such as *Streptococcus* comprise both commensal and opportunistic lineages (Xie et al., 2024). Accordingly, our inference of virulence potential is framed at the level of community-distributed functional programmes (e.g., adhesion, invasion, immune-interface modulation) rather than assuming uniform pathogenicity of any given genus. This distinction mitigates over-interpretation of taxonomic signals and reinforces the ecological principle that virulence emerges from coordinated functional organisation across communities, not from the presence of individual “pathogenic” taxa alone.

However, oral microbiome profiles derived from saliva, oral rinse, mucosal swabs, plaque-associated material, or tissue-derived specimens are not directly interchangeable, because each captures different microbial niches and therefore different ecological structure. Likewise, variation in 16S region selection, sequencing platform, denoising pipeline, taxonomic reference framework, and HPV detection methodology can materially alter downstream diversity estimates, compositional profiles, and inferred coordination patterns. For this reason, cross-cohort pooling was not attempted in the present study. Although pooling may appear to increase sample size, combining cohorts with heterogeneous sampling frames and ascertainment strategies would risk introducing artefactual ecological gradients and obscuring the community-level signal under investigation. Accordingly, the present findings should be interpreted within the analytical context of the source dataset and validated prospectively across harmonized oral HPV-microbiome cohorts.

Key strengths of our study include;

- The use of public bacterial genomics data leveraging high-quality 16S rRNA sequencing data from NCBI SRA ensures transparency, reproducibility and scalability.
- Ecology-first conceptual study that analysed oral HPV-microbiome interactions from taxon-centric associations to community-level ecological virulence organisation, addressing a central limitation of prior work.
- It provides new biological insights grounded explanation for why previous studies reported heterogeneous and inconsistence taxonomic signatures despite consistent HPV-microbiome associations.
- The integration of virology and bacterial genomics anchored in HPV epithelial biology and microbial functional organisation, rather than purely statistical correlations is novel.
- Ecological virulence modelling interrogating taxa-virulence coordination and functional coupling, the study captures higher-order microbiome structure invisible to standard differential abundance analyses studies.
- Separation of virulence load from virulence organisation is conceptually novel and biologically insightful.
- Unsupervised ecotype analysis showed that HPV does not define microbiome ecotypes strengthens causal interpretation and rules out trivial clustering effects.
- Variance partitioning (PERMANOVA) clearly established ecological resilience and virulence structure as dominant axes over HPV status alone.
- Clear translational relevance by identifying ecological and network-level features with potential utility for risk stratification and intervention beyond single-taxon biomarkers.

Limitations and their justifications include;

- Cross-sectional study design which represents a single time point and conflates transient infection with persistence. Longitudinal sampling is required to disentangle acquisition, clearance and persistence, but such data are not available in the public SRA resource analysed.
- 16S rRNA gene sequencing resolution, species- and strain-level resolution as well as direct measurement of virulence genes, is limited. Shotgun metagenomics or meta-transcriptomics would be required but were not available for this cohort and would substantially reduce sample size and comparability.
- Lack of direct host immune or epithelial measurements. Host immunity, epithelial integrity and mucosal inflammation are inferred ecologically rather than measured directly. These data are not captured in the original sequencing study and cannot be retrospectively generated.
- Only binary HPV status and does not capture viral load, transcriptional activity or specific HPV genotypes, all of which influence persistence biology. Such virological granularity was not available in the source dataset.
- Virulence modules were inferred from curated bacterial genomics databases rather than measured experimentally. This is an inherent limitation of amplicon-based studies but remains standard and biologically interpretable at population scale (Waseem et al., 2017).
- Potential residual confounding from behavioural and oral health variables (smoking, periodontal disease severity, sexual behaviour) are incompletely captured. However, the ecological framework explicitly accommodates upstream heterogeneity by focusing on community organisation rather than single covariates (Gilbert and Lynch, 2019).
- Generalisability across populations. Findings derive from this cohort and may vary in magnitude across populations. Nonetheless, the ecological principles identified are expected to generalise because they are independent of specific taxa (Foster et al., 2017; Gilbert and Lynch, 2019).
- Reliance on a single publicly available cohort. Upon PRISMA screening, this study was based on re- analysis of a single oral HPV-microbiome dataset, which limits cross-cohort validation. Multi-cohort integration was not pursued because substantial heterogeneity in sampling sites, sequencing protocols and HPV ascertainment could obscure higher-order ecological signals when pooled naively. Accordingly, this work is positioned as framework-defining and hypothesis-generating rather than a population-level synthesis, with independent validation across additional cohorts warranted.
- We did not address tumour microenvironment biology, lesion progression, or cancer outcomes due to the nature of the available data.

However, these limitations are intrinsic to publicly available, cross-sectional bacterial genomics data and do not undermine the central ecological and mechanistic conclusions of this study.

## Conclusions

This study shows that oral HPV infection does not define a discrete bacterial microbiome state nor drive dominant taxonomic restructuring. Instead, it aligns with pre-existing, low-resilience oral microbial communities organised through coordinated virulence ecology. HPV infection aligned with structured rewiring of ecological virulence relationships particularly those governing epithelial adhesion, invasion and immune-interface functions. These findings support the hypothesis that HPV persistence is a context-dependent of bacterial microbiome ecological virulence phenomenon, shaped by microbial community organisation and resilience rather than by individual bacterial taxa. Translationally, this work may establish a framework for microbiome-informed risk stratification biomarkers for oral HPV persistence and cancer progression. However, the current defined ecological framework requires validation in longitudinal, multi-cohort, and clinically phenotyped datasets. Our future study will include host oropharynx mucosal immunity and higher-resolution metagenomics to validate the oncogenic risk.

## Data Availability

The original contributions presented in the study are included in the article/**Supplementary Material**. Further inquiries can be directed to the corresponding author.
