# Supplementary figures and images for: Oral human papillomavirus infection aligns with a coordinated bacterial microbiome inferred virulence ecology
Source: Front Cell Infect Microbiol. 2026 Jun 5;16:1821266. doi: 10.3389/fcimb.2026.1821266 (PMC13279419; doi:10.3389/fcimb.2026.1821266)

$\Delta$  taxa–virulence coordination (HPV+ – HPV–)  
Spearman  $\rho$  difference per genus  $\times$  module

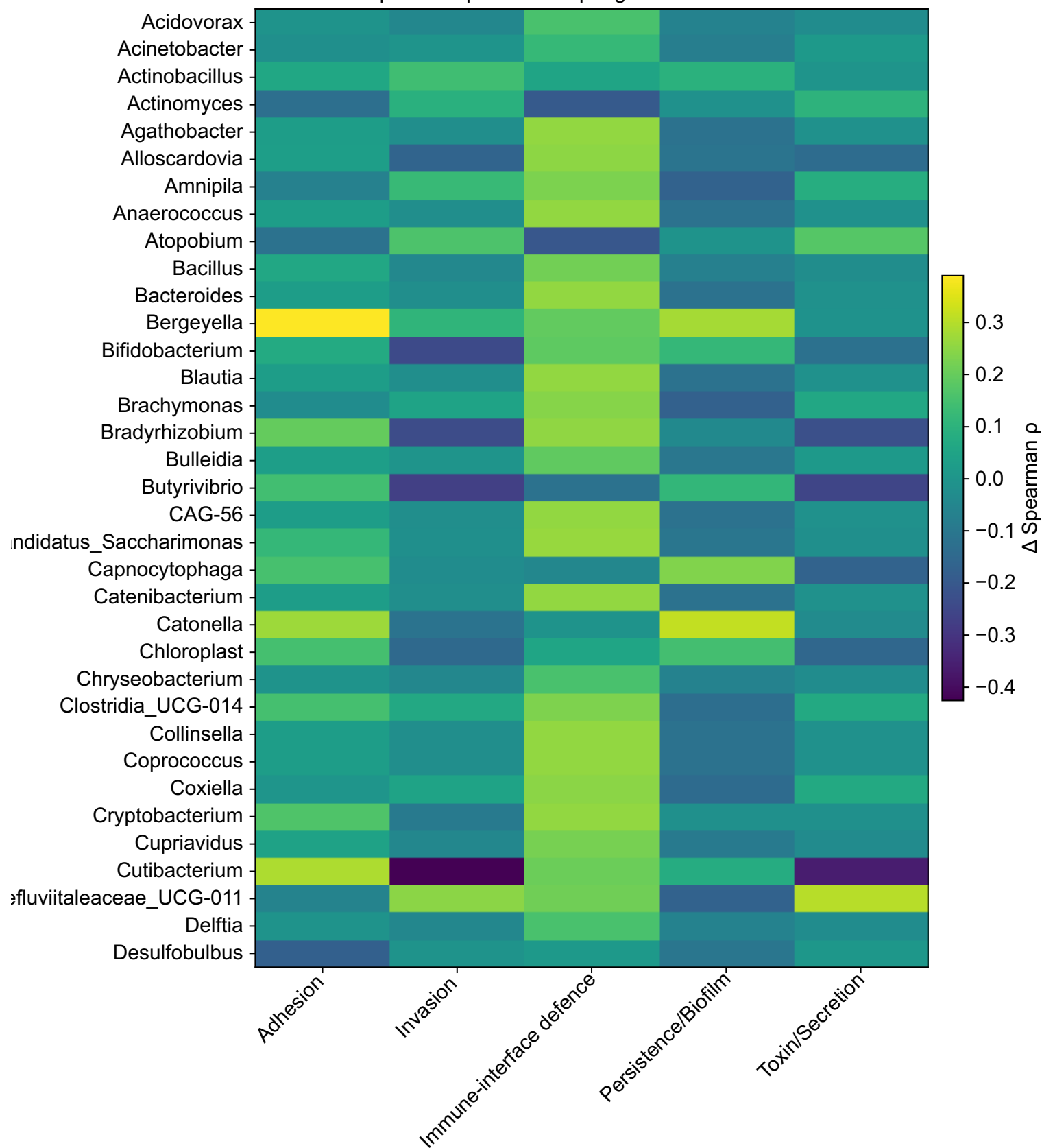

Supplement: Supplementary file 1 [file DataSheet1.pdf]

# Genus-level abundance by HPV status

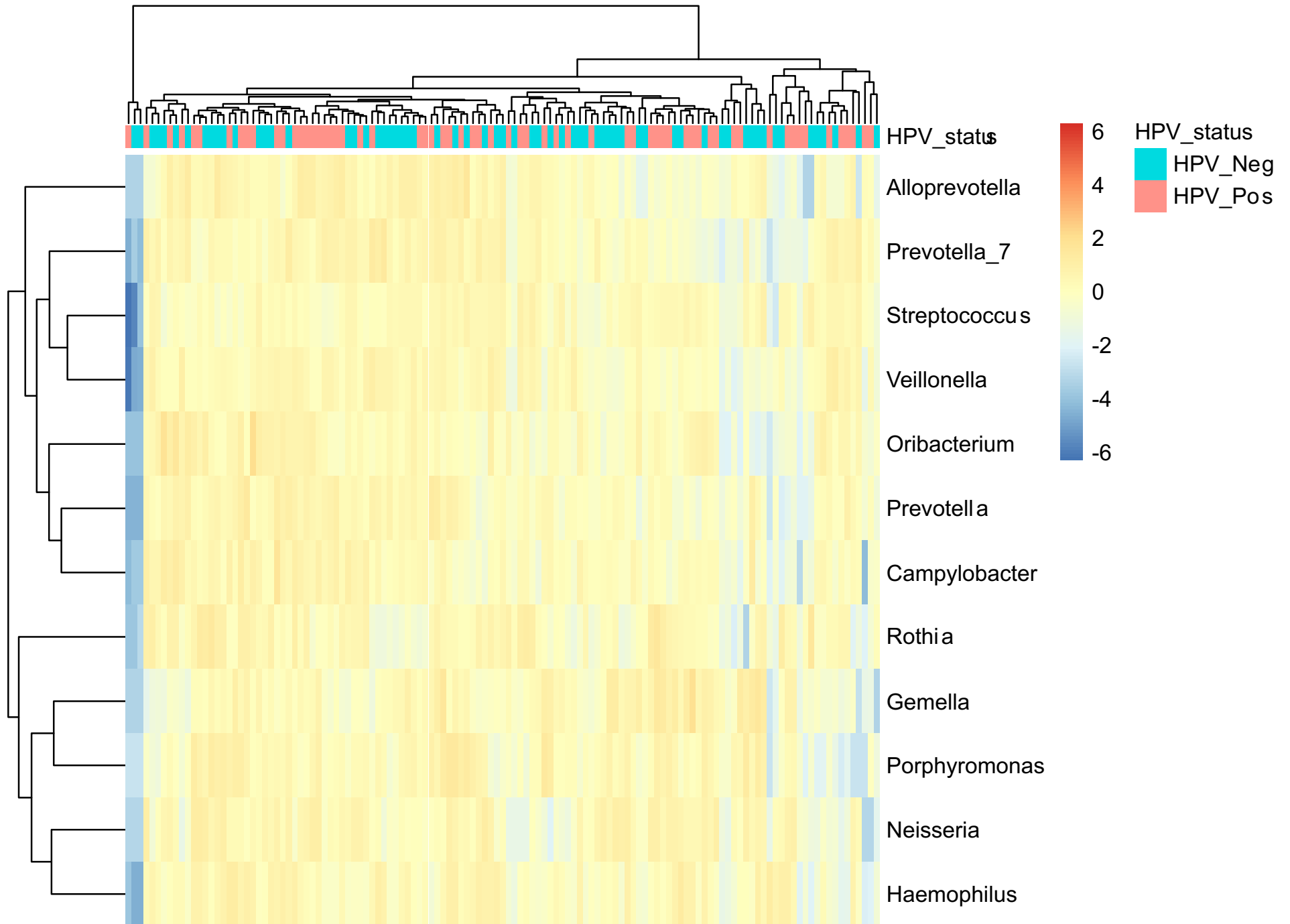

Supplement: Supplementary file 2 [file DataSheet2.pdf]

a.

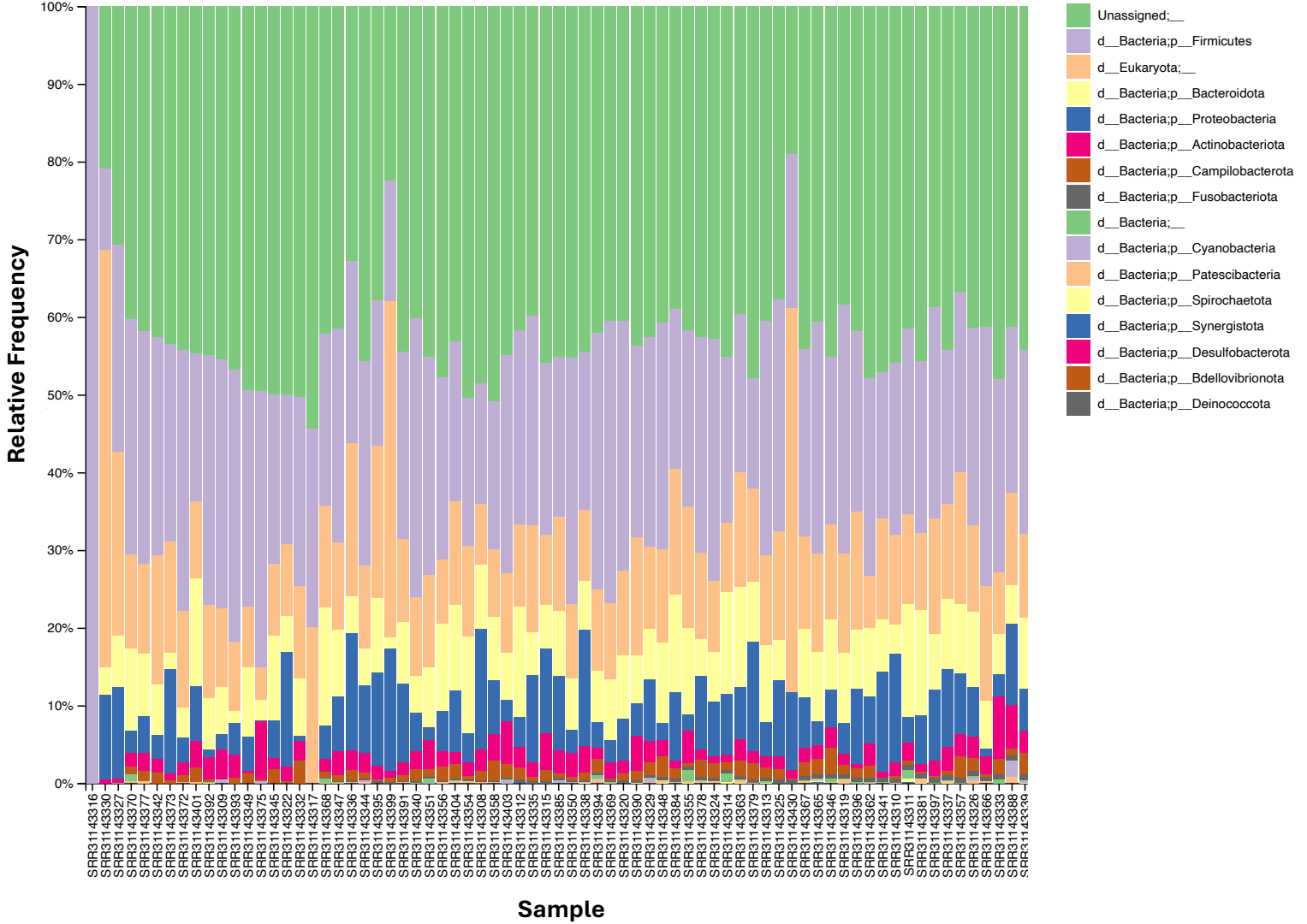

b.

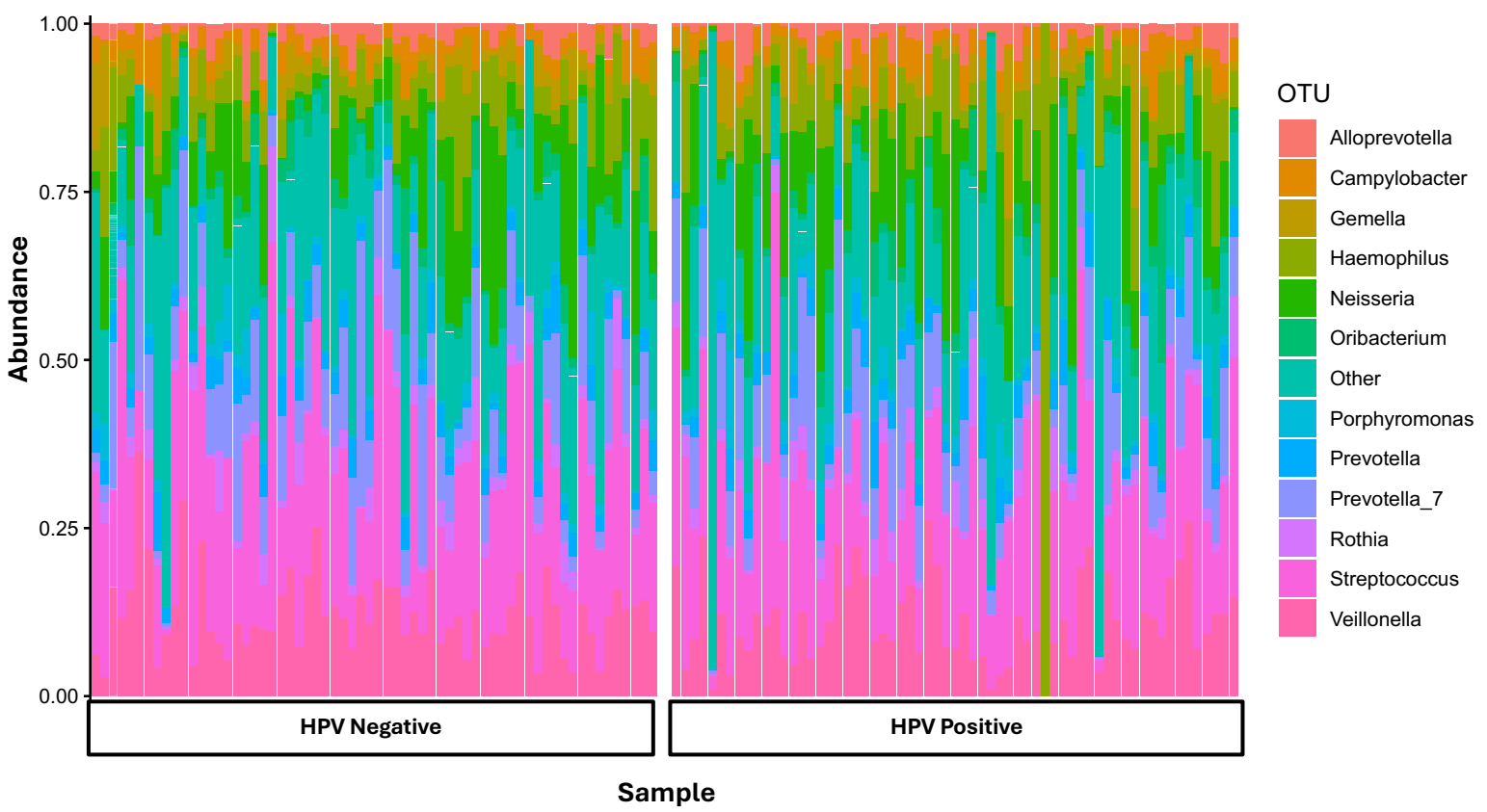

Supplement: Supplementary file 3 [file DataSheet3.pdf]

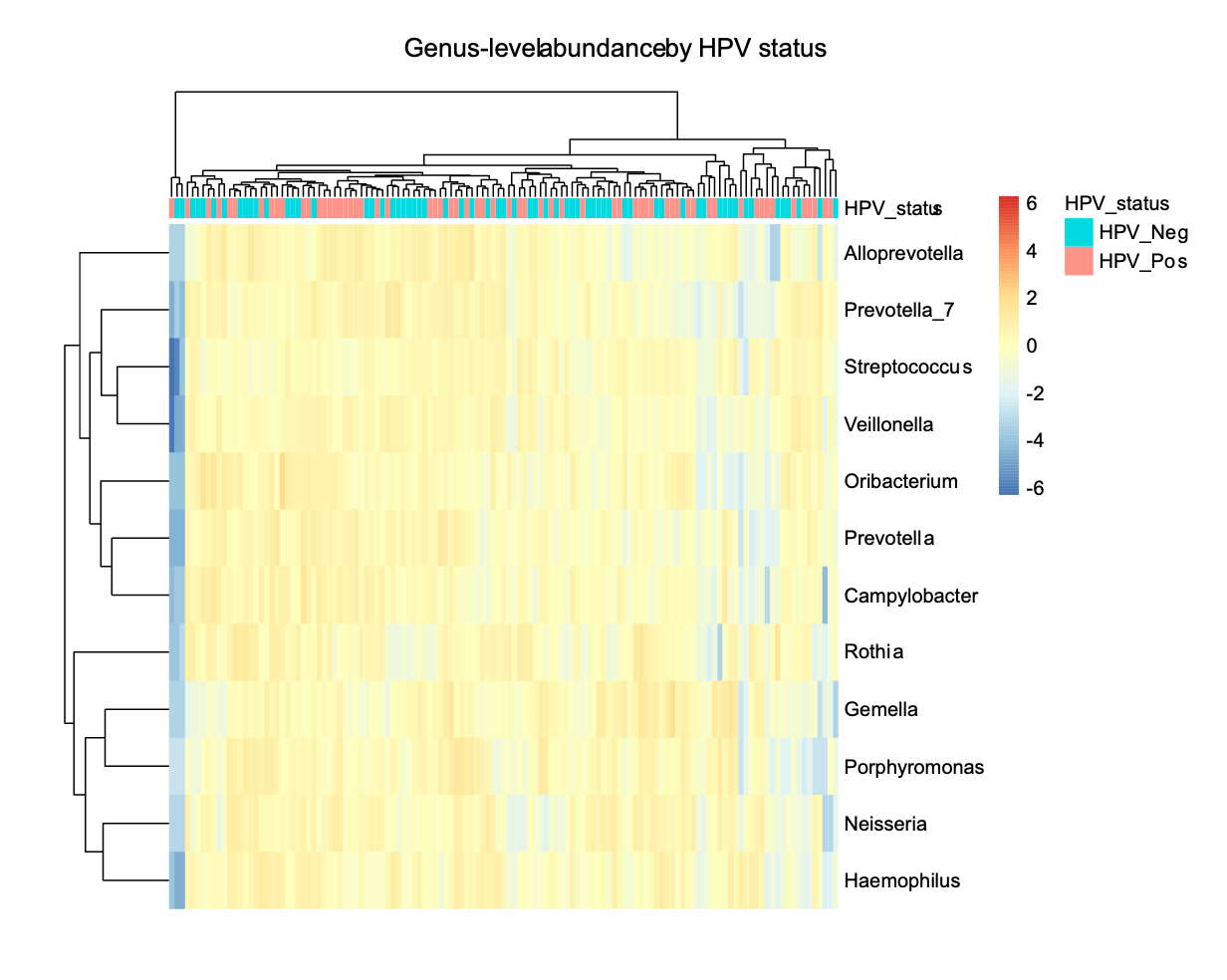

Supplement: Supplementary file 16 [file Table1.docx]
