# Supplementary material for: Oral human papillomavirus infection aligns with a coordinated bacterial microbiome inferred virulence ecology
Source: Front Cell Infect Microbiol. 2026 Jun 5;16:1821266. doi: 10.3389/fcimb.2026.1821266 (PMC13279419; doi:10.3389/fcimb.2026.1821266)
Supplement: Supplementary file 10 [file DataSheet10.pdf]

**Supplementary Methods S2 Sensitivity and robustness analyses**

**Overview**

To ensure that the observed associations between oral HPV infection and virulence-structured microbiome ecology were not driven by analytical artefacts, taxonomic sparsity, or modelling assumptions, we implemented a comprehensive sensitivity and robustness framework spanning annotation strategy, compositional handling, network inference, and statistical modelling.

**Robustness to virulence annotation strategy**

Virulence domain assignments were stress-tested using alternative curation sources. Primary analyses based on VFDB-aligned virulence categories were re-evaluated using:

(i) VFDB-only annotations,

(ii) VFDB combined with Victors, and

(iii) VFDB combined with BV-BRC pathogenomics annotations.

Across all annotation strategies, the direction and relative magnitude of virulence coordination signals were preserved, indicating that results were not dependent on any single database or curation schema. Genera with ambiguous or context-dependent virulence evidence were iteratively excluded, and core findings remained stable, demonstrating conservative inference.

**Drop-one-domain sensitivity analysis**

To exclude the possibility that results were driven by a single virulence category, composite virulence ecology metrics were recalculated after sequentially removing each domain (adhesion, invasion, immune-interface modulation, biofilm persistence, toxin/secretion, and exoenzyme activity). Removal of any single domain did not abolish the observed HPV-associated ecological patterns, confirming that virulence coordination emerged as a distributed, multi-domain property rather than a category-specific artefact.

**Compositional data robustness**

All primary analyses were performed on centred log-ratio (CLR)–transformed genus-level data to address the compositional nature of 16S rRNA sequencing. Sensitivity analyses using alternative normalisation approaches yielded concordant qualitative results, indicating that network-level coordination patterns were not driven by spurious correlations arising from library size or relative abundance constraints. Correlation analyses were restricted to positive associations, and multiple-testing correction was applied using the Benjamini–Hochberg procedure.

**Network inference stability**

Taxa–virulence coordination networks were reconstructed across a range of correlation thresholds and false-discovery rate cut-offs. Core conclusions regarding structured edge rewiring and module-specific coordination

were robust to these parameter changes. Edge accumulation and rewiring patterns were consistent when restricting analyses to high-prevalence genera, demonstrating that results were not driven by sparse or low-abundance features.

**Permutation and resampling analyses**

To assess the likelihood of observing the reported coordination patterns by chance, HPV labels were permuted across samples and network metrics recalculated. Observed HPV-associated coordination exceeded the distribution obtained under permutation, supporting a non-random ecological association. Bootstrap resampling was additionally applied to cumulative coordination analyses to assess variability and confidence in curve separation.

**Multivariate model robustness**

Variance partitioning results were confirmed across alternative PERMANOVA model specifications and distance metrics. In all models, microbial diversity and virulence ecology explained a substantially larger fraction of community variation than HPV status alone, supporting the conclusion that HPV aligns with pre-existing ecological structure rather than acting as a dominant community driver.

**Interpretive boundaries**

All sensitivity analyses were interpreted within the predefined scope of genus-level functional potential. No claims were made regarding strain-specific virulence gene presence, expression, or causality. Collectively, these robustness assessments demonstrate that the central findings are stable across analytical choices and reflect genuine ecological organisation rather than methodological artefacts.

**References**


1. Matchado MS, Rühlemann MC, Reitmeier S, et al. *On the limits of 16S rRNA gene-based metagenome prediction and functional profiling.* Microbial Genomics. 2024;10(2):001203. doi:10.1099/mgen.0.001203.
2. Liu B, Zheng D, Zhou S, Chen L, Yang J. *VFDB 2022: a general classification scheme for bacterial virulence factors.* Nucleic Acids Res. 2022;50(D1):D912-D917. doi:10.1093/nar/gkab1107.
3. Gloor GB, Macklaim JM, Pawlowsky-Glahn V, Egozcue JJ. *Microbiome datasets are compositional: and this is not optional.* Front Microbiol. 2017;8:2224. doi:10.3389/fmicb.2017.02224.
4. Douglas GM, Maffei VJ, Zaneveld J, et al. *PICRUSt2: An improved and customizable approach for metagenome inference.* Nat Biotechnol. 2020;38(6):685-688. doi:10.1038/s41587-020-0548-6.
5. Zhou H, He K, Chen J, et al. *LinDA: Linear models for differential abundance analysis of microbiome compositional data.* Genome Biol. 2022;23:95. doi:10.1186/s13059-022-02655-5.
