# Supplementary material for: Oral human papillomavirus infection aligns with a coordinated bacterial microbiome inferred virulence ecology
Source: Front Cell Infect Microbiol. 2026 Jun 5;16:1821266. doi: 10.3389/fcimb.2026.1821266 (PMC13279419; doi:10.3389/fcimb.2026.1821266)
Supplement: Supplementary file 11 [file DataSheet11.pdf]

Supplementary Methods S5.

Construction of virulence ecology metrics and composite scores

Conceptual rationale

Virulence ecology metrics were constructed to quantify distributed, coordinated functional organisation of the oral microbiome rather than taxon-specific virulence burden, enabling detection of HPV-associated ecological alignment. The central aim of this study was not to quantify virulence burden as a simple sum of taxa-associated functions, but to capture higher-order ecological organisation of virulence potential within the oral microbiome. In microbial ecosystems, virulence-relevant traits are frequently distributed across taxa and exert biological effects through coordination and co-expression rather than dominance of individual organisms. Accordingly, we developed composite virulence ecology metrics that integrate both the distribution and the coordination of virulence-associated functions across the bacterial community.

Taxonomic assignment

Taxonomic classification of ASVs was performed with a dual-database strategy to maximise species-level accuracy in the oral microbiome. The expanded Human Oral Microbiome Database (eHOMD v15.22) was selected as the primary reference due to its curated, habitat-specific taxonomy designed specifically for oral and aerodigestive tract microbiomes. Representative ASV sequences generated by DADA2 were first aligned against eHOMD using QIIME2's feature-classifier classify-sklearn algorithm with a pre-trained Naïve Bayes classifier built on the eHOMD V3–V4 region. The classifier's k-mer-based probabilistic model allowed high-precision assignments while minimising misclassifications among closely related oral taxa. Taxonomic confidence scores were inspected to ensure robust classification depth at genus and species levels. To validate assignments and capture taxa that may be underrepresented or absent in eHOMD, a secondary classification was performed against the Genome Taxonomy Database (GTDB release R207). GTDB's genome-based phylogeny enables high-resolution taxonomic placement and provides an orthogonal confirmation layer, particularly for uncultured or candidate phyla organisms frequently detected in oral microbiomes (e.g., Saccharibacteria/TM7, Gracilibacteria/GN02).

Concordance between eHOMD and GTDB annotations was evaluated; in cases of disagreement, eHOMD was prioritised for named oral species, while GTDB classifications were retained for higher-level taxonomy or unassigned oral lineages. This hybrid approach ensured maximal biological fidelity, enabling us to reliably identify pathogenic and oncogenic taxa of interest such as *Porphyromonas gingivalis*, *Fusobacterium nucleatum*, *Prevotella spp.*, *Parvimonas micra*, *Sneathia spp.*, *Bacteroidales G-2*, and other taxa previously implicated in epithelial disruption, inflammation, and cancer-promoting microbial activities.

A final curated taxonomy table was generated after removing non-bacterial contaminants (mitochondrial and chloroplast ASVs) and collapsing rare ASVs where necessary for downstream ecological and functional analyses. This table formed the basis for the targeted pathogenic/oncogenic microbial profiling and virulence-linked functional characterisation central to the study's aims.

Virulence domain abundance scores

For each sample, virulence domain abundance scores were computed by aggregating the CLR-transformed abundances of genera annotated to a given VFDB-aligned virulence domain. Aggregation was performed using the arithmetic mean of CLR values within each domain, ensuring that scores reflected relative enrichment or depletion rather than absolute abundance. This approach preserves compositional validity and avoids inflation by highly abundant single taxa. To facilitate comparison across domains, domain-level scores were z-standardised across the full cohort. These normalised values represent relative virulence potential within each domain and were used exclusively for comparative and network analyses.

Composite virulence ecology score

To summarise multi-domain virulence organisation at the community level, we constructed a Composite Virulence Ecology Score (VES). The VES was calculated as the mean of the z-standardised domain scores across all virulence domains retained after quality filtering. All domains contributed equally, and no domain-specific weighting was applied, reflecting the absence of a priori assumptions regarding domain dominance. This unweighted formulation was selected to ensure interpretability and robustness, and to emphasise distributed virulence architecture rather than amplification of specific functional categories. Sensitivity analyses confirmed that alternative weighting schemes did not materially alter conclusions.

Virulence coordination metrics

To capture functional organisation beyond abundance, we quantified virulence coordination by evaluating correlations between genus-level abundances and virulence domain scores across samples. Spearman rank correlations were computed between each genus and each virulence domain score, and associations were retained following false discovery rate correction. For each sample group, a coordination density metric was derived by counting the number of statistically significant genus–domain associations, reflecting the degree of ecological coupling between taxa and virulence functions. Additionally, cumulative coordination curves were generated by ranking genera according to their alignment with the composite virulence score and computing the accumulation of significant coordination edges across this ranked axis.

Virulence rewiring and comparative metrics

Differences in virulence organisation between HPV-positive and HPV-negative samples were quantified using comparative coordination metrics, including differences in coordination density and area under the coordination accumulation curve (ΔAUC). These measures capture rewiring of functional relationships rather than shifts in virulence load and are therefore insensitive to changes in overall abundance. Permutation testing was employed to assess statistical significance, with HPV labels randomly reassigned to samples and ΔAUC recalculated to generate empirical null distributions.

Interpretive scope and limitations

All virulence ecology metrics quantify genus-level functional potential and coordination, not gene-level presence or expression. The metrics are designed to detect ecological alignment and organisation, consistent with the

study's hypothesis that oral HPV infection aligns with pre-existing virulence-structured microbial states rather than inducing discrete compositional changes.

References

1. Escapa IF, Chen T, Huang Y, et al. New insights into human nostril microbiome from the expanded Human Oral Microbiome Database (eHOMD): a resource for the microbiome of the human aerodigestive tract. mSystems. 2018;3(6):e00187-18. doi:10.1128/mSystems.00187-18.

2. F. Escapa, I., Huang, Y., Chen, T. *et al.* Construction of habitat-specific training sets to achieve species-level assignment in 16S rRNA gene datasets. *Microbiome* **8**, 65 (2020). https://doi.org/10.1186/s40168-020-00841-w

3. Callahan BJ, McMurdie PJ, Rosen MJ, et al. DADA2: high-resolution sample inference from Illumina amplicon data. Nat Methods. 2016;13(7):581-583. doi:10.1038/nmeth.3869.

4. Bokulich NA, Kaehler BD, Rideout JR, et al. Optimizing taxonomic classification of marker-gene amplicon sequences with QIIME 2's q2-feature-classifier plugin. Microbiome. 2018;6(1):90. doi:10.1186/s40168-018-0470-z.

5. Parks DH, Chuvochina M, Rinke C, et al. GTDB release 10: a complete and systematic taxonomy for Bacteria and Archaea. Nucleic Acids Res. 2025;gkaf1040. doi:10.1093/nar/gkaf1040.

6. Le, F, Dumuid, D. Stanford, T. E., & Wiley, J. F. (2025). Bayesian Multilevel Compositional Data Analysis with the R Package *multilevelcoda*. *Multivariate Behavioral Research*, 1–19. https://doi.org/10.1080/00273171.2025.2565598.

7. Gloor GB, Macklaim JM, Pawlowsky-Glahn V, et al. Microbiome datasets are compositional: and this is not optional. *Front Microbiol.* 2017;8:2224. doi:10.3389/fmicb.2017.02224.

8. Weiss S, Van Treuren W, Lozupone C, et al. Correlation detection strategies in microbial data sets vary widely in sensitivity and precision. *ISME J.* 2016;10(7):1669–1681. doi:10.1038/ismej.2015.235.

9. Layeghifard M, Hwang DM, Guttman DS. Disentangling interactions in the microbiome: a network perspective. *Trends Microbiol.* 2017;25(3):217–228. doi:10.1016/j.tim.2016.11.008.

11. Langille MGI, Zaneveld J, Caporaso JG, et al. Predictive functional profiling of microbial communities using 16S rRNA marker gene sequences. *Nat Biotechnol.* 2013;31(9):814–821. doi:10.1038/nbt.2676.

12. Douglas GM, Maffei VJ, Zaneveld JR, et al**.** PICRUSt2 for prediction of metagenome functions. *Nat Biotechnol.* 2020;38(6):685–688. doi:10.1038/s41587-020-0548-6

13. Liu B, Zheng D, Jin Q, Chen L, Yang J. VFDB 2022: a general classification scheme for bacterial virulence factors. *Nucleic Acids Res.* 2022;50(D1):D912–D917. doi:10.1093/nar/gkab1107

14. Sayers S, Li L, Ong E, Deng S, et al. Victors: a web-based knowledge base of virulence factors in human and animal pathogens. *Nucleic Acids Res.* 2019;47(D1):D693–D700. doi:10.1093/nar/gky999

15. Yost S, Stashenko P, Choi Y, Kukuruzinska M, Genco CA, et al. Increased virulence of the oral microbiome in oral squamous cell carcinoma revealed by metatranscriptome analyses. *Int J Oral Sci.* 2018;10:32. doi:10.1038/s41368-018-0037-7
