# Supplementary material for: Oral human papillomavirus infection aligns with a coordinated bacterial microbiome inferred virulence ecology
Source: Front Cell Infect Microbiol. 2026 Jun 5;16:1821266. doi: 10.3389/fcimb.2026.1821266 (PMC13279419; doi:10.3389/fcimb.2026.1821266)
Supplement: Supplementary file 12 [file DataSheet12.pdf]

Supplementary Methods S1

Systematic identification of eligible oral HPV-microbiome sequencing datasets

Rationale for systematic dataset identification

Secondary genomic data analyses are increasingly used to address mechanistic hypotheses that could not be resolved within single primary studies. However, such analyses are vulnerable to selection bias if dataset identification is not conducted transparently and systematically. To ensure reproducibility, minimise bias, and align with best practices for secondary data research, we implemented a PRISMA-guided workflow for dataset identification framework to systematically identify publicly available oral microbiome sequencing datasets suitable for investigating the ecological virulence architecture associated with oral human papillomavirus (HPV) infection. Although the final analysis focused on a single dataset, this outcome reflects the application of predefined, biologically and analytically motivated eligibility criteria rather than post hoc dataset selection. The systematic workflow ensures that the included dataset represents the only currently available cohort capable of supporting the specific ecological and virulence-based analyses undertaken in this study.

Databases and data sources searched

A comprehensive search was conducted across genomic sequence repositories and biomedical literature databases that index human microbiome studies with associated phenotype metadata. Searches were restricted to databases that provide access to raw sequencing data and sample-level annotations.

Databases searched included:

(i) NCBI Sequence Read Archive (SRA)
(ii) NCBI BioProject
(iii) ENA

Search strategy and query design

The search strategies were designed to identify studies jointly profiling oral bacterial microbiomes and oral HPV infection status. Boolean operators were applied to ensure sensitivity while maintaining topical specificity.

Core search terms included:
(i) “oral microbiome” AND “HPV”
(ii) “oral bacterial microbiome” AND “human papillomavirus”
(iii) “oral 16S” AND “HPV”
(iv) “oropharyngeal microbiome” AND “HPV infection”

For NCBI SRA and BioProject, keyword searches were supplemented by manual screening of associated metadata fields, including *sample description*, *study design*, and *phenotype annotations*. Reference lists of eligible studies were manually screened to identify additional datasets not captured through automated searches (snowballing).

Eligibility criteria

Eligibility criteria were defined a priori and applied uniformly at the dataset level. Criteria were selected to ensure biological relevance, analytical compatibility, and methodological robustness for virulence-ecology inference.

Inclusion criteria

Datasets were eligible for inclusion if they met all of the following criteria:

(i) Human oral microbiome samples
- Samples derived from the oral cavity, oropharynx, or closely related mucosal sites.

(ii) Adult participants (≥18 years)
- Pediatric cohorts were excluded to avoid confounding by developmental microbiome dynamics.

(iii) 16S rRNA gene sequencing
- Marker-gene sequencing enabling genus-level ecological and functional inference.

(iv) Sample-level oral HPV status available
- HPV phenotype reported per individual sample.

(v) Public access to raw sequencing data with clinical metadata
- Availability of FASTQ files with clinical metadata to enable independent reprocessing and compositional analysis.

(vi) Adequate sequencing depth and quality
- Suitable for diversity, ordination, and network-based analyses after quality control.

Exclusion criteria

Datasets were excluded if any of the following applied:

(i) Pediatric or mixed pediatric–adult cohorts
- Excluded to prevent age-dependent ecological confounding.

(ii) Absence of oral HPV data
- Studies reporting HPV prevalence at cohort level only, without sample-level data, were excluded.

(iii) Lack of raw sequencing data
- Studies providing only processed tables or summary statistics were excluded.

(iv) Incomplete or inconsistent metadata
- Datasets lacking key covariates required for analysis.

Dataset screening and selection process

All records identified through database searches were screened sequentially:

(i) Title and abstract screening to exclude clearly irrelevant studies.
(ii) Metadata inspection to assess availability of HPV phenotype and sample-level annotations.
(iii) Full-text and repository review to confirm sequencing type, cohort characteristics, and raw data accessibility.

Screening decisions were documented and exclusion reasons were recorded to ensure transparency. This process culminated in the identification of a single dataset that satisfied all eligibility criteria.

Final included dataset

Only one publicly available dataset met all predefined inclusion criteria:

(i) Adult oral microbiome cohort
(ii) 16S rRNA gene sequencing
(iii) Sample-level oral HPV status
(iv) Complete raw sequencing data
(v) Sufficient clinical metadata

This dataset was therefore included for secondary genomic analysis and forms the basis of all downstream results presented in this study.

Summary of eligibility framework

Category	Criterion	Justification
Population	Adults only (≥18 years)	To avoid developmental microbiome confounding
Body site	Oral / oropharyngeal samples	For biological relevance to oral HPV
Sequencing	16S rRNA gene	To enable genus-level ecological inference
HPV data	Sample-level HPV status	To exposure-microbiome alignment
Data access	Raw FASTQ files	To have reproducible secondary analysis
Metadata	Age, HPV status	For ecological modelling and adjustment

Methodological Implications

This systematic approach ensures that the dataset used in this study was not selected opportunistically but represents the only fully accessed currently available cohort capable of supporting virulence-ecology-focused analyses of oral HPV infection. Our framework provides a reproducible template for future secondary genomic studies as additional datasets become available.

References

1. Page MJ, McKenzie JE, Bossuyt PM, et al. The PRISMA 2020 statement: an updated guideline for reporting systematic reviews. BMJ. 2021;372:n71. doi:10.1136/bmj.n71.
2. Page MJ, Moher D, Bossuyt PM, et al. PRISMA 2020 explanation and elaboration: updated guidance and exemplars for reporting systematic reviews. BMJ. 2021;372:n160. doi:10.1136/bmj.n160.
3. Mirzayi C, Renson A, Zohra F, et al. Reporting guidelines for human microbiome research: the STORMS checklist. Nat Med. 2021;27(11):1885-1892. doi:10.1038/s41591-021-01552-x.
4. Wilkinson MD, Dumontier M, Aalbersberg IJ, et al. The FAIR Guiding Principles for scientific data management and stewardship. Sci Data. 2016;3:160018. doi:10.1038/sdata.2016.18.
5. Bolyen E, Rideout JR, Dillon MR, et al. Reproducible, interactive, scalable and extensible microbiome data science using QIIME 2. Nat Biotechnol. 2019;37(8):852-857. doi:10.1038/s41587-019-0209-9.
6. Callahan BJ, McMurdie PJ, Rosen MJ, et al. DADA2: High-resolution sample inference from Illumina amplicon data. Nat Methods. 2016;13(7):581-583. doi:10.1038/nmeth.3869.
7. Sinha R, Abu-Ali G, Vogtmann E, et al. Assessment of variation in microbial community amplicon sequencing by the Microbiome Quality Control (MBQC) project consortium. Nat Biotechnol. 2017;35(11):1077-1086. doi:10.1038/nbt.3981.
8. Yilmaz P, Kottmann R, Field D, et al. Minimum information about a marker gene sequence (MIMARKS) and minimum information about any (x) sequence (MIxS) specifications. Nat Biotechnol. 2011;29(5):415-420. doi:10.1038/nbt.1823.
9. Leinonen R, Sugawara H, Shumway M, et al. The sequence read archive. Nucleic Acids Res. 2011;39(Database issue):D19-D21. doi:10.1093/nar/gkq1019.
10. von Elm E, Altman DG, Egger M, et al. The Strengthening the Reporting of Observational Studies in Epidemiology (STROBE) statement: guidelines for reporting observational studies. PLoS Med. 2007;4(10):e296. doi:10.1371/journal.pmed.0040296.
