# Supplementary material for: Oral human papillomavirus infection aligns with a coordinated bacterial microbiome inferred virulence ecology
Source: Front Cell Infect Microbiol. 2026 Jun 5;16:1821266. doi: 10.3389/fcimb.2026.1821266 (PMC13279419; doi:10.3389/fcimb.2026.1821266)

PRISMA flow diagram for identification of oral HPV–microbiome sequencing datasets

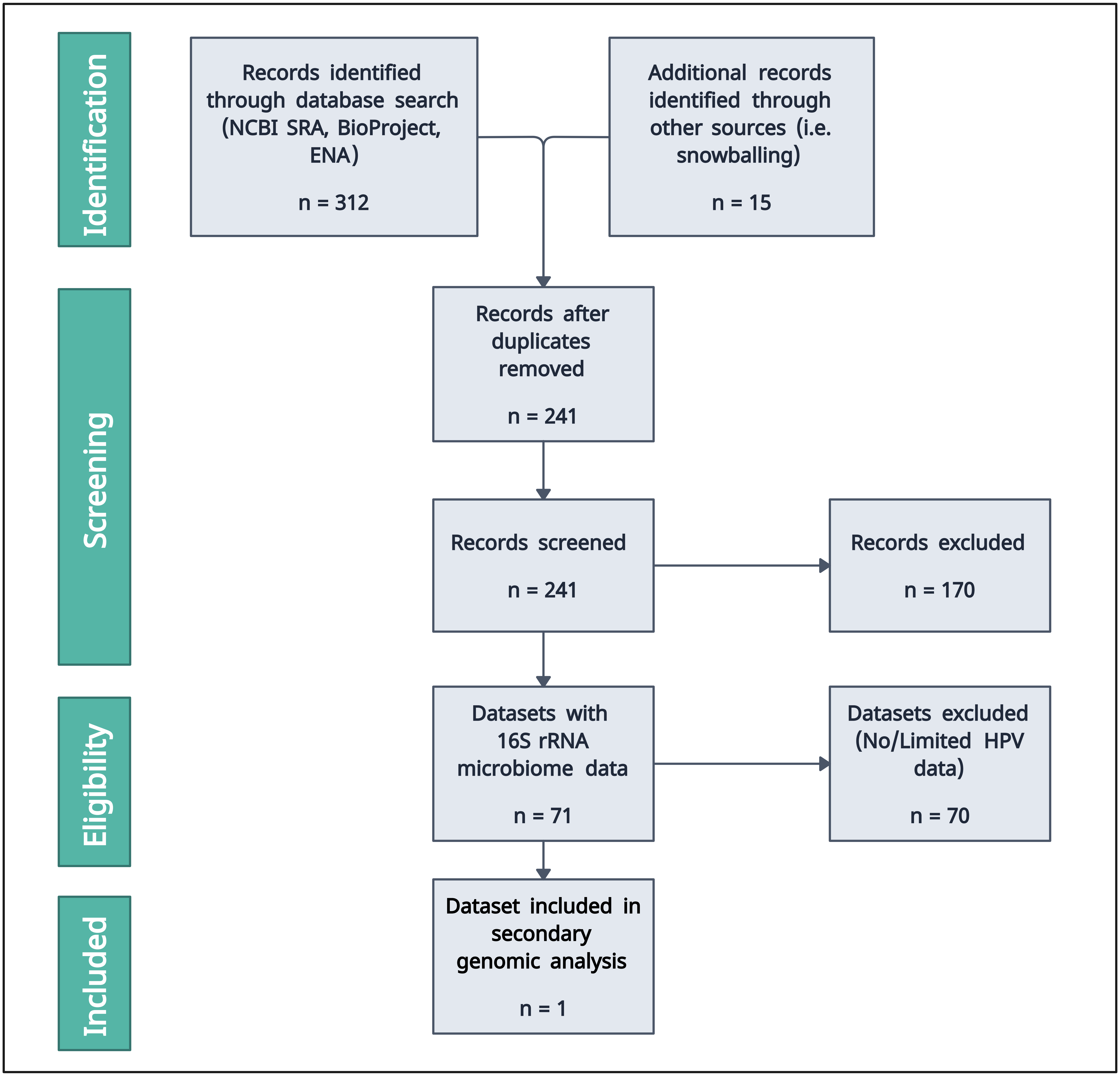

Supplement: Supplementary file 13 [file DataSheet13.pdf]
