# Supplementary material for: Oral human papillomavirus infection aligns with a coordinated bacterial microbiome inferred virulence ecology
Source: Front Cell Infect Microbiol. 2026 Jun 5;16:1821266. doi: 10.3389/fcimb.2026.1821266 (PMC13279419; doi:10.3389/fcimb.2026.1821266)
Supplement: Supplementary file 25 [file Table10.docx]

############################################################

# ALL PLOTS ‚Äî NO FactoMineR ‚Äî SAVE AS PDF

############################################################

# -------------------------

# Libraries (base-safe)

# -------------------------

library(ggplot2)

library(pheatmap)

library(igraph)

library(reshape2)

# -------------------------

# Load data

# -------------------------

pathway <- read.table(

"metacyc_pathways.tsv",

header = TRUE,

sep = "\t",

row.names = 1,

check.names = FALSE

)

meta <- read.table(

"metadata_clean.tsv",

header = FALSE,

sep = "\t",

stringsAsFactors = FALSE

)

colnames(meta) <- c(

"SampleID","HPV_status","Sex","Age","Smokestat","alcohol_2cat"

)

taxa <- read.table(

"taxa_export/taxa_table.tsv",

header = TRUE,

sep = "\t",

row.names = 1,

comment.char = "#", # üî¥ FIXED

check.names = FALSE

)

# -------------------------

# Matrices

# -------------------------

taxa_mat <- as.matrix(taxa)

pathway_mat <- as.matrix(pathway)

# -------------------------

# Match samples (CORRECT AXES)

# -------------------------

common_samples <- Reduce(

intersect,

list(

colnames(taxa_mat), # taxa: samples = columns

rownames(pathway_mat), # MetaCyc: samples = rows

meta$SampleID

)

)

# -------------------------

# Prepare matrices

# -------------------------

taxa_m <- taxa_mat[, common_samples, drop = FALSE]

path_m <- pathway_mat[common_samples, , drop = FALSE]

path_m <- t(path_m) # pathways as rows, samples as columns

taxa_m <- taxa_m[apply(taxa_m, 1, var) > 0, , drop = FALSE]

path_m <- path_m[apply(path_m, 1, var) > 0, , drop = FALSE]

ann <- factor(meta$HPV_status[match(common_samples, meta$SampleID)])

############################################################

# 1Ô∏è‚É£ VIOLIN PLOT (SAFE FALLBACK)

############################################################

# ===============================

# Violin plot: MetaCyc pathway vs HPV

# ===============================

library(ggplot2)

# Load data

pathway <- read.table(

"metacyc_pathways.tsv",

header = TRUE, sep = "\t",

row.names = 1, check.names = FALSE

)

meta <- read.table(

"metadata_clean.tsv",

header = FALSE, sep = "\t",

stringsAsFactors = FALSE

)

# Assign correct metadata headers

colnames(meta) <- c(

"SampleID",

"HPV_status",

"Sex",

"Age",

"Smokestat",

"alcohol_2cat"

)

# Match samples safely

common_samples <- intersect(colnames(pathway), meta$SampleID)

# Select most variable MetaCyc pathway

top_pwy <- names(

which.max(apply(pathway[, common_samples], 1, var))

)

# Build plotting dataframe

df <- data.frame(

HPV_status = factor(

meta$HPV_status[match(common_samples, meta$SampleID)],

levels = c("HPV negative", "HPV positive")

),

Abundance = as.numeric(pathway[top_pwy, common_samples])

)

# ===============================

# Plot + Save as PDF

# ===============================

pdf("Fig_MetaCyc_Violin_HPV.pdf", width = 6, height = 5)

ggplot(df, aes(x = HPV_status, y = Abundance, fill = HPV_status)) +

geom_violin(trim = FALSE, alpha = 0.85, color = "black") +

geom_boxplot(

width = 0.15,

outlier.shape = NA,

fill = "white",

color = "black"

) +

scale_fill_manual(

values = c("HPV negative" = "#F8766D",

"HPV positive" = "#00BFC4")

) +

theme_bw(base_size = 12) +

theme(

legend.title = element_blank(),

legend.position = "right",

axis.title.x = element_blank(),

axis.text.x = element_text(size = 11),

axis.title.y = element_text(size = 11),

plot.title = element_text(face = "bold", hjust = 0.5)

) +

labs(

y = paste0("Predicted abundance (", top_pwy, ")"),

title = "Top variable MetaCyc pathway by HPV status"

)

dev.off()

############################################################

# 2Ô∏è‚É£ HEATMAP ‚Äî Top 50 MetaCyc pathways

############################################################

library(pheatmap)

# Load data

pathway <- read.table(

"metacyc_pathways.tsv",

header = TRUE, sep = "\t",

row.names = 1, check.names = FALSE

)

meta <- read.table(

"metadata_clean.tsv",

header = FALSE, sep = "\t",

stringsAsFactors = FALSE

)

# Fix metadata headers

colnames(meta) <- c(

"SampleID",

"HPV_status",

"Sex",

"Age",

"Smokestat",

"alcohol_2cat"

)

# Match samples

common_samples <- intersect(colnames(pathway), meta$SampleID)

# Select TOP 50 most variable pathways

var_rank <- apply(pathway[, common_samples], 1, var)

top_pwy <- names(sort(var_rank, decreasing = TRUE))[1:50]

# Build matrix

mat <- pathway[top_pwy, common_samples]

mat <- log10(mat + 1)

# Column annotation

ann_col <- data.frame(

HPV_status = factor(

meta$HPV_status[match(common_samples, meta$SampleID)],

levels = c("HPV negative", "HPV positive")

)

)

rownames(ann_col) <- common_samples

ann_colors <- list(

HPV_status = c(

"HPV negative" = "#F8766D",

"HPV positive" = "#00BFC4"

)

)

# BEAUTIFUL color palette

heat_cols <- colorRampPalette(

c("#313695", "#74ADD1", "#FFFFBF", "#FDAE61", "#A50026")

)(100)

# VIEW heatmap

pheatmap(

mat,

annotation_col = ann_col,

annotation_colors = ann_colors,

clustering_method = "ward.D2",

scale = "row",

color = heat_cols,

show_colnames = FALSE,

fontsize_row = 6,

border_color = NA

)

pdf("Fig_MetaCyc_Heatmap_Top50_HPV.pdf", width = 8, height = 10)

pheatmap(

mat,

annotation_col = ann_col,

annotation_colors = ann_colors,

clustering_method = "ward.D2",

scale = "row",

color = heat_cols,

show_colnames = FALSE,

fontsize_row = 6,

border_color = NA

)

dev.off()

############################################################

# 3Ô∏è‚É£ PCA ‚Äî BASE R (prcomp)

############################################################

library(ggplot2)

library(FactoMineR)

# PCA (already computed, but safe to recompute)

X <- t(mat)

pca_res <- PCA(X, scale.unit = TRUE, graph = FALSE)

# Build plotting dataframe

pca_df <- data.frame(

PC1 = pca_res$ind$coord[,1],

PC2 = pca_res$ind$coord[,2],

HPV_status = ann_col$HPV_status

)

# % variance explained

pc1_var <- round(pca_res$eig[1,2], 1)

pc2_var <- round(pca_res$eig[2,2], 1)

# Plot

ggplot(pca_df, aes(PC1, PC2, color = HPV_status)) +

geom_point(size = 3, alpha = 0.8) +

stat_ellipse(level = 0.95, linewidth = 1) +

scale_color_manual(

values = c("HPV negative" = "#F8766D",

"HPV positive" = "#00BFC4")

) +

theme_bw() +

labs(

x = paste0("PC1 (", pc1_var, "%)"),

y = paste0("PC2 (", pc2_var, "%)"),

title = "PCA of predicted MetaCyc pathways"

)

pdf("Fig_MetaCyc_PCA_HPV.pdf", width = 6, height = 5)

ggplot(pca_df, aes(PC1, PC2, color = HPV_status)) +

geom_point(size = 3, alpha = 0.8) +

stat_ellipse(level = 0.95, linewidth = 1) +

scale_color_manual(

values = c("HPV negative" = "#F8766D",

"HPV positive" = "#00BFC4")

) +

theme_bw() +

labs(

x = paste0("PC1 (", pc1_var, "%)"),

y = paste0("PC2 (", pc2_var, "%)"),

title = "PCA of predicted MetaCyc pathways"

)

dev.off()

############################################################

# 4Ô∏è‚É£ TAXA √ó PATHWAY CORRELATION HEATMAP

############################################################

top_taxa <- rownames(taxa_m)[order(apply(taxa_m,1,var), decreasing=TRUE)][1:20]

top_path <- rownames(path_m)[order(apply(path_m,1,var), decreasing=TRUE)][1:20]

cor_mat <- cor(

t(taxa_m[top_taxa, ]),

t(path_m[top_path, ]),

method="spearman"

)

cor_mat[is.na(cor_mat)] <- 0

pdf("Fig4_Taxa_Pathway_Correlation_Heatmap.pdf", 8, 6)

pheatmap(

cor_mat,

color=colorRampPalette(c("navy","white","firebrick3"))(100),

fontsize_row=7,

fontsize_col=7,

border_color=NA,

main="Taxa‚ÄìVirulence Pathway Co-variation"

)

dev.off()

############################################################

# 5Ô∏è‚É£ NETWORK ‚Äî TAXA‚ÄìPATHWAY INTERACTIONS

############################################################

edges <- which(abs(cor_mat) > 0.6, arr.ind=TRUE)

edge_df <- data.frame(

from=rownames(cor_mat)[edges[,1]],

to=colnames(cor_mat)[edges[,2]],

weight=cor_mat[edges]

)

g <- graph_from_data_frame(edge_df, directed=FALSE)

pdf("Fig5_Taxa_Pathway_Network.pdf", 7, 7)

plot(

g,

vertex.size=6,

vertex.label.cex=0.6,

edge.width=abs(E(g)$weight)*2,

edge.color=ifelse(E(g)$weight>0,"firebrick","steelblue"),

main="Taxa‚ÄìVirulence Pathway Interaction Network"

)

dev.off()

############################################################

# END ‚Äî ALL FIGURES SAVED

############################################################
